# Biobased Polyamides: A Journey from the Biomass Towards Cutting Edge Materials

**DOI:** 10.3390/polym17192599

**Published:** 2025-09-25

**Authors:** Maria Diaz-Galbarriatu, Julia Sánchez-Bodón, Estíbaliz Hernáez-Laviña, José Luis Vilas-Vilela, Isabel Moreno-Benítez

**Affiliations:** 1 Innovative Macromolecular Materials (IMACROMAT), Department of Physical Chemistry, UPV/EHU, 48940 Leioa, Spain; mariadiazg@ehu.eus (M.D.-G.); julia.sanchez@ehu.eus (J.S.-B.); estibaliz.hernaez@ehu.eus (E.H.-L.); joseluis.vilas@ehu.eus (J.L.V.-V.); 2 BCMaterials, Basque Centre for Materials, Applications and Nanostructures, UPV/EHU Science Park, 48940 Leioa, Spain; 3 Innovative Macromolecular Materials (IMACROMAT), Department of Organic and Inorganic Chemistry, UPV/EHU, 48940 Leioa, Spain

**Keywords:** polyamides, biobased, furan, terpene, fatty acids

## Abstract

Since the 1930s, polyamides (PAs) have become increasingly vital across industries like automotive, textiles, electronics, and packaging, owing to their exceptional properties. However, they also have notable limitations, including a tendency to absorb water, low dimensional stability, poor solubility, and the resulting processing challenges. From an environmental perspective, the reliance on fossil-based monomers for traditional PAs and the accumulation of post-consumer waste, due to their resistance to (bio)degradation, are key concerns. In recent decades, significant advancements have been made in synthesizing PAs from bio-based monomers, primarily sourced from inedible lignocellulosic materials. Some of these bio-based PAs exhibit properties comparable to their fossil-derived counterparts, with benefits like enhanced solubility, which simplifies processing. Moreover, certain bio-based variants have shown improved biodegradability, facilitating the potential recovery of monomers for the production of new virgin polymers and reducing waste accumulation. This review highlights the progress in developing PAs from commonly used bio-based sources, including lignin-derived aromatic compounds, terpenes, fatty acids, and furan derivatives, with a focus on the improvements made over their fossil-based analogs.

## 1. Introduction

Polyamides (PAs), as the name suggests, constitute a class of polymer characterized by the presence of amide bonds between repeating units [1,2,3]. This structural feature enables the formation of hydrogen bonds between adjacent polymeric chains [1,4,5], thus conferring upon these materials outstanding mechanical properties as well as high chemical resistance [6,7,8,9]. For that reason, since the development of the aliphatic PA Nylon 6,6 and Nylon 6 [2,6,10], first synthesized by Carothers at DuPont and Paul T. Schlack, respectively, in the 1930s [11], these materials have found widespread use across a diverse range of applications. These include consumer products as well as high-performance smart materials in sectors such as electronics, textiles, automotive or medicine [8,12,13]. However, PAs also present notable drawbacks. For instance, their tendency to absorb water considerably limits their dimensional stability [14]. Indeed, the absorbed water acts as a plasticizer and, consequently, worsens both mechanical and thermal properties. On the other hand, the same strong intermolecular interactions that provide the previously mentioned desirable attributes, such as high strength and chemical resistance, also lead to very high melting points, usually inaccessible, elevated transition temperatures and poor solubility in common organic solvents. These characteristics make the processing of certain PAs, particularly aromatic ones, extremely challenging and economically costly, thereby limiting their high-scale production [3]. Consequently, over the last 20 years, many semi-aromatic PAs have been developed to combine the excellent thermal, mechanical, and chemical properties of aromatic PAs with the easier processing of aliphatic ones [4,14].

**Scheme 1:**
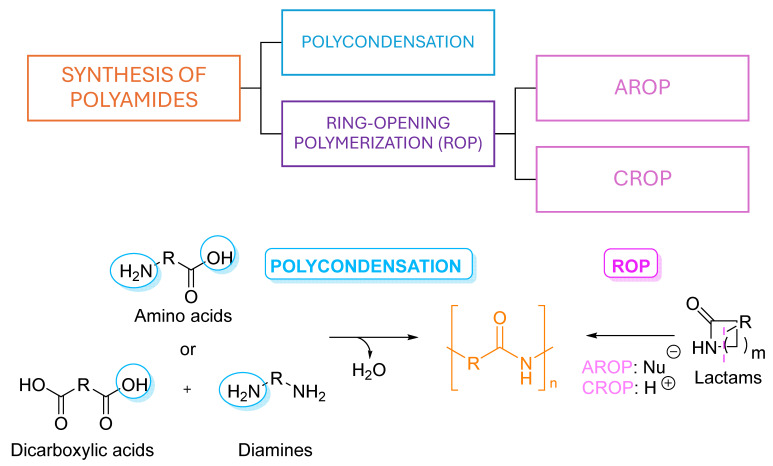
General approaches for the synthesis of PAs: polycondensation reaction between diacids and diamines or aminoacids, and ring-opening polymerization starting from lactams.

There are two common approaches for synthesizing PAs (Scheme 1): The first method is the step-growth polycondensation employing diamines and dicarboxylic acids, diesters or diacyl chlorides [15,16]. Alternatively, ω-amino acids or esters can be used as the only substrates in this polymerization. The second methodology, commonly employed to prepare PAs, is the ring-opening polymerization (ROP) of lactams [1,3,17]. For instance, ԑ-caprolactam is the most well-known lactam for making Nylon 6 through anionic ring-opening polymerization (AROP) [15].

In any case, the starting substrates used in both synthetic methodologies have traditionally been obtained from the petrochemical industry [18]. However, the limited availability and the volatility of the prices of fossil resources are the main reasons that have encouraged industry and academia to look for other alternatives [3,19]. Notably, in recent years, real efforts have been made to develop polymers from more renewable feedstocks that maintain the excellent properties of their petro-based counterparts while minimizing the environmental impact [20,21,22]. Moreover, the incorporation of renewable feedstocks in polymer synthesis significantly enhances the biodegradability of the resulting materials, thereby mitigating their long-term persistence in the environment and reducing their contribution to environmental pollution [23,24]. Additionally, these materials often demonstrate a reduced carbon footprint over their entire life cycle, as the biomass used in their production sequesters atmospheric CO_2_ during growth. This carbon uptake partially compensates for the greenhouse gas emissions associated with subsequent stages such as manufacturing, processing, and transportation [8,25,26].

This review aims to provide a comprehensive overview of the path from the first steps in the synthesis of PAs that presented mediocre properties very far from the fossil-derived analogues to the current state in which promising innovative materials have been achieved with excellent properties, high proportions of biogenic carbon and great (bio)degradability.

## 2. Synthesis of PAs from Lignin-Derived Monomers

**Figure 1:**
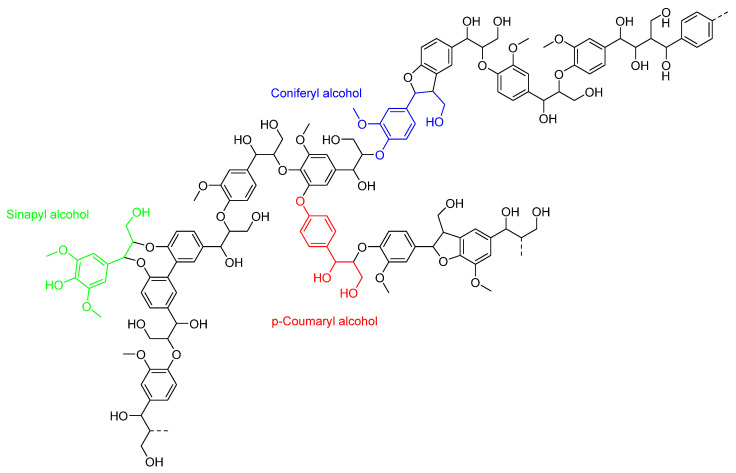
Chemical structure of lignin.

Lignin is the largest non-carbohydrate component of lignocellulosic biomass. Effectively, after cellulose, this biopolymer constitutes the second most abundant renewable polymer. The oxidative coupling of three aromatic monomers, *p*-coumaryl, coniferyl, and sinapyl alcohols in different proportions depending on the source, originates the complex and amorphous highly branched network characteristic of lignin (Figure 1) [3]. Recently, lignin and its derivatives have emerged as potential components for diverse polymer applications, such as flame retardants [27], antimicrobial agents [28], stabilizing agents [29], coatings [30], and superabsorbent hydrogels [31,32], owing to the high carbon content, good thermal stability, biodegradability, and antioxidant/antibacterial activity [33].

**Figure 2:**
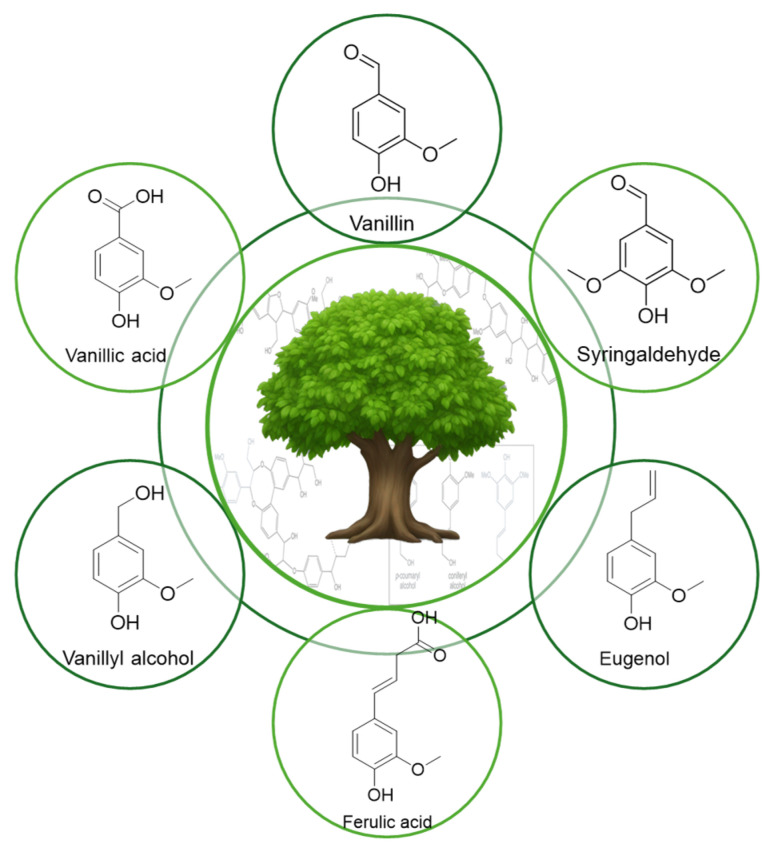
Lignin aromatic derivatives structures.

Despite extensive research into controlled depolymerization of lignin, the efficient and economically viable production of aromatic compounds from this abundant biopolymer remains limited [34,35]. Indeed, its complex structure, strong bonds, unpredictable decomposition products, and variability are some of the reasons why valorizing lignin is so extremely difficult [36]. To date, only a few aromatic compounds, such as guaiacol, eugenol, ferulic acid, vanillin and syringaldehyde, can be obtained from lignin using economical and effective methodologies (see Figure 2) [34,35]. These aromatic compounds have been employed as monomers for the synthesis of various biobased polymers [36]. This review will specifically focus on the synthesis of PAs derived from the aromatic compounds, vanillin, eugenol and ferulic acid.

### 2.1. Vanillin-Based PAs

The lignin-derived aromatic compound vanillin holds significant potential as a precursor to prepare biobased monomers owing to the chemical versatility of its carbonyl and phenolic functional groups [37]. However, it is noteworthy that the polymers obtained from this monomer usually have relatively low molecular weights, mainly due to the low reactivity of this aromatic derivative [38].

**Scheme 2:**
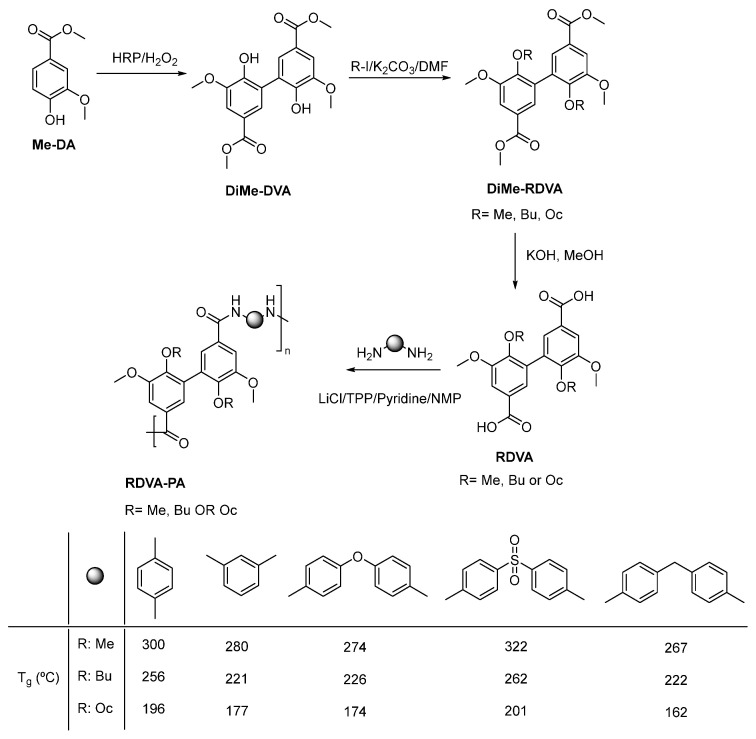
Synthesis of divanillic acid-derived monomers (RDVA) and subsequent polymerization with structurally different diamines, as well as the transition temperatures (T_g_) of the so-obtained polymers.

In 2021, K. Yagura et al. [39] reported the synthesis of a series of biobased PAs through a polycondensation protocol using different diamines and divanillic acid (DVA) previously obtained across an enzymatic process (Scheme 2). In this case, the obtained polymers presented very high weight-average molecular weights (M_w_) ranging from 50 to 110 kDa. They observed that the use of the dimeric starting compound not only enhanced the molecular weights of the polymers but also improved their solubility. Indeed, the vast majority of the synthesized PAs were highly soluble in common organic solvents, which allowed the successful preparation of cast films and thermo-pressed films. These findings highlight DVAPAs as promising high-performance bio-based plastics with superior thermal properties compared to many other renewable aromatic polymers. The tensile strengths of the prepared polymers ranged from 30 MPa to 60 MPa, depending on the length of the lateral substituent, and were, therefore, as strong as polystyrene or common polycarbonate.

**Scheme 3:**
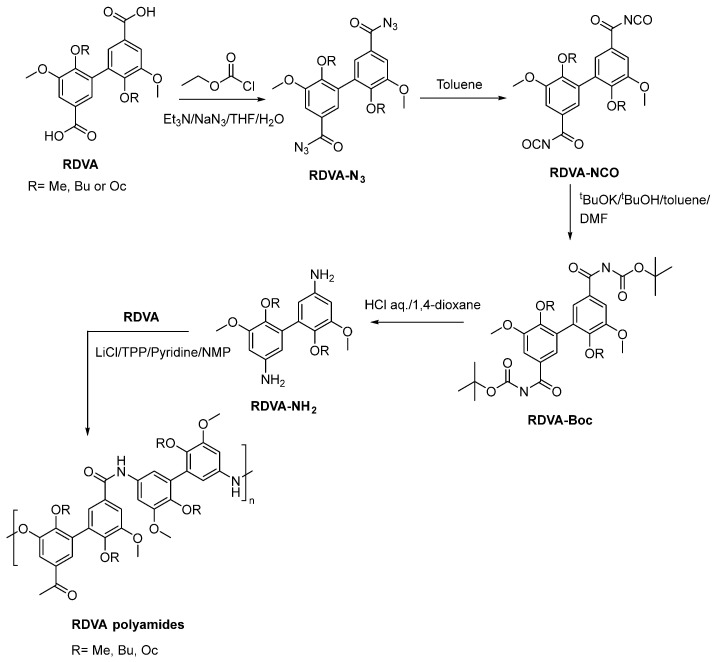
Synthesis of fully DVA-derived PAs by reacting RDVA monomer with RDVA-NH_2_.

Subsequently, these same authors synthesized fully DVA-PAs with M_w_ ranging from 49 to 60 kDa, reacting RDVA monomer with RDVA-NH_2_, a diamine monomer derived from RDVA (Scheme 3). Thermogravimetric analysis of the PAs found decomposition temperatures around 380 °C. The T_g_ values of fully divanillic DVAPAs ranged from 207 °C to 262 °C, depending on the side chain length. Notably, DVAPAs with shorter side chains exhibited higher T_g_ values than those with longer side chains [40]. The maximum tensile strength achieved with these materials was 40 MPa, suggesting a certain analogy in terms of mechanical properties to polyesterene.

**Scheme 4:**
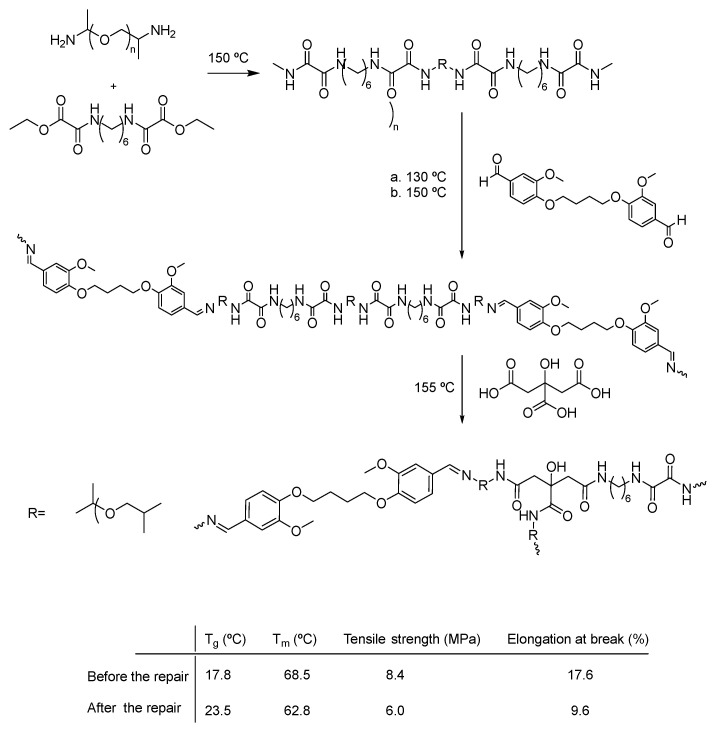
Synthesis of a vanillin-based poly(amide-imide) vitrimer starting from a prepolymer synthesized through an acylation reaction and DVA.

Similarly and more recently, Y. Zhao et al. described the synthesis of a vanillin-based poly(amide-imide) vitrimer. For this purpose, a NH_2_-terminated PA was prepared through an acylation process. Subsequently, this prepolymer was reacted with previously synthesized DVA monomer (Scheme 4). This vanillin-based polymer presented excellent stability, and the amide and imine dynamic bonds conferred healing performance, showing similar mechanical and thermal properties before and after the repair. In addition to the partially biobased origin, the authors also demonstrated the recyclability of the material, making it an even more attractive option for environmentally conscious applications [41].

### 2.2. Eugenol-Based PAs

Another promising building block that can be obtained through lignin depolymerization is eugenol, although, currently, it is predominantly extracted from clove oil. This natural compound possesses suitable and reactive functional groups for chain growth polymerization, including allylic double bond, as well as additional reactive sites, such as the aromatic ring, hydroxyl and methoxy groups [25,42].

**Scheme 5:**
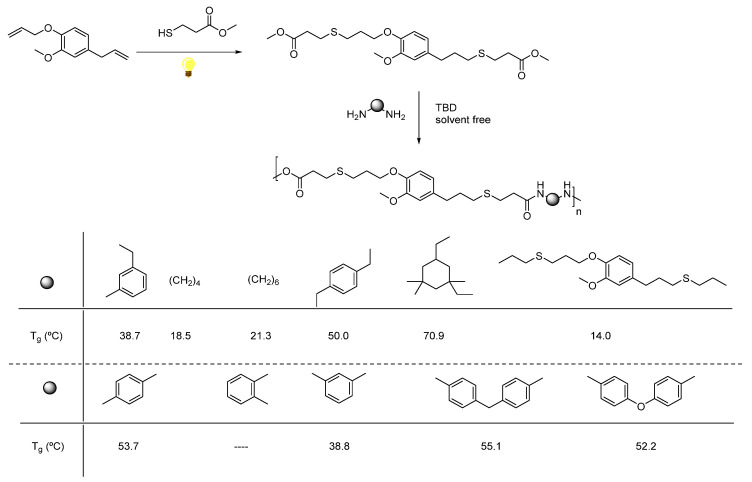
Solvent-free polycondensation reaction of eugenol-derived diester monomer and a series of aliphatic and aromatic diamines for the synthesis of amorphous semiaromatic PAs.

Although different polymers have been synthesized from this natural compound to the best of our knowledge, to date, the work recently published by our research group constitutes the only one related to the synthesis of PAs derived from this natural phenolic compound. In this paper, the synthesis of a wide series of semiaromatic sulfur-containing PAs was presented. For that purpose, starting from eugenol, a diester monomer was easily synthesized and subsequently polycondensed with a number of structurally diverse aliphatic and aromatic diamines in order to study the scope of the methodology and the influence of the diamine counterpart on the properties of the final polymer. All the prepared materials resulted in amorphous materials showing excellent thermal properties and dimensional stability. In addition to the bio-based nature of the materials, other aspects such as the solvent-free process and the employment of an eco-friendly catalyst are also noteworthy from an environmental point of view (Scheme 5) [43].

### 2.3. Ferulic Acid-Based PAs

**Scheme 6:**
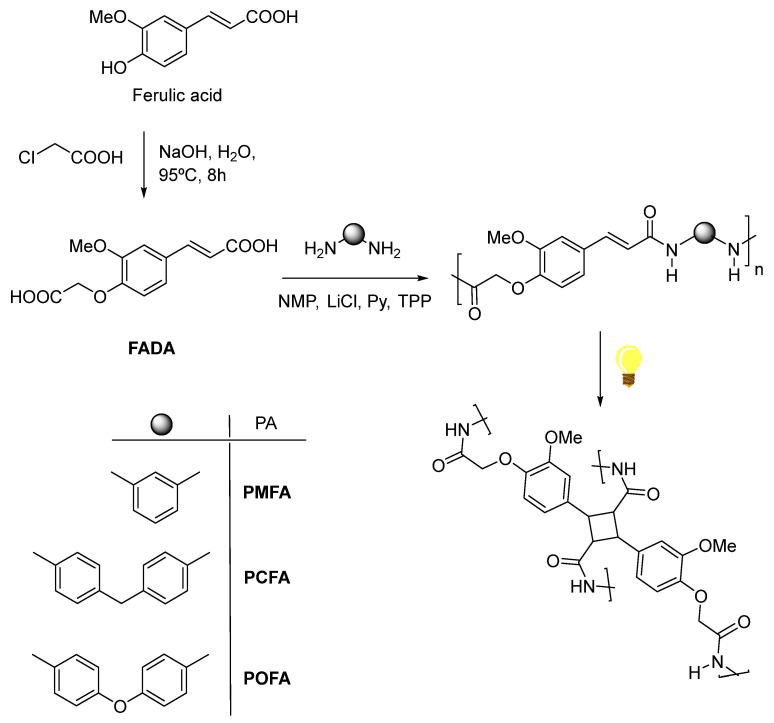
Synthesis of **FADA**-derived PAs and photoinduced cross-linking through [2+2] cyclization.

Ferulic acid is another aromatic lignin-derived hydroxycinnamic acid, very abundant in the plant kingdom. It is bound to lignin through covalent ester or ether bonds and is usually efficiently obtained by basic hydrolysis processes. The α,β-unsaturated carboxylic acid and phenol functional groups present in this natural compound have allowed different research groups to use it as a building block in the synthesis of polymers [44]. Regarding PAs, recently, Z. Tang and collaborators have described the successful preparation of a series of PAs from FADA, an aromatic diacid easily accessible from ferulic acid, using the Yamazaki-Higashi phosphorylation protocol. The so-obtained materials presented high molecular weights (M_n_ > 32,000), narrow dispersities (1.7–2.1) and excellent thermal stabilities (T_d_^5%^ > 300 °C). Moreover, the synthesized polymers were soluble in common aprotic solvents, which greatly facilitates their processability, typically very problematic with aromatic PAs. In addition, these PAs also exhibited excellent thermal processing; indeed, uniform films were successfully obtained at 200–300 °C. An exhaustive study of the mechanical properties of the three newly synthesized materials revealed tensile strength of 40–80 MPa, tensile modulus of 2000 MPa, and an elongation at break of 10–15%. Compared to the previously mentioned materials derived from vanillic acid, FADA-based PA exhibited better mechanical properties under similar molecular weights, with the added advantage that access to the monomer from this natural source is much faster and more effective. Furthermore, both the thermal and mechanical properties were further improved by photoinduced crosslinking through [2+2] cyclizations of the internal olefinic groups. Moreover, these authors employed quantum chemical simulations to carry out a complete theoretical study on the chemical recycling of these materials. Calculations revealed that the lowest energy barrier corresponded to the PMFA PA, which was corroborated by experimental tests. Indeed, excellent recovery rates of the starting monomer were achieved when PAs were treated in a basic aqueous solution. Interestingly, degradation experiments of the cross-linked samples were also successful, although more drastic reaction conditions were necessary (Scheme 6) [45]. 

To conclude this section, it can be stated that vanillin, eugenol, and ferulic acid, three natural compounds derived from lignin, have proven to be effective starting materials for synthesizing new PAs. The aromatic structure of these compounds imparts excellent properties to the resulting PAs, often on par with some fossil-derived counterparts. However, challenges persist. As noted, all the polyamides discussed here have been synthesized through polycondensation reactions, and in most cases, one of the monomers is still derived from fossil sources. Additionally, the synthetic processes required to obtain the actual monomers from natural sources are, in some instances, quite lengthy. Therefore, it would be advisable to design fast and efficient synthetic routes to obtain monomers from natural substrates, avoiding the use of additional petroleum-derived products that would lower the biogenic carbon content in the final products.

## 3. Furan-Based PAs

**Figure 3:**
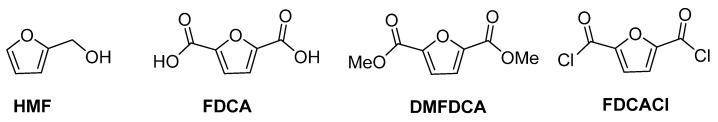
Furanic compounds.

In 2004, the US Department of Energy published the Platform Chemicals List, which was later updated in 2010. This is a list of organic compounds containing from 2 to 6 carbon atoms that can be obtained by catalytic or biocatalytic processes from the lignocellulosic part of biomass [46]. It is hoped that these biobased building blocks will provide an opportunity to replace fossil sources for obtaining energy and chemical products. Two furanic compounds, 5-hydroxymethylfurfural (HMF) and 2,5-furandicarboxylic acid (FDCA) (see Figure 3), were added to the revised list. Indeed, it is believed that these heterocyclic aromatic compounds are the ones with the greatest synthetic potential. Moreover, the scientific community has accepted these derivatives as key elements in bridging the gap between a fossil fuel-based economy and a sustainable one, due to the similar or better properties of the biobased materials, showing similar thermal properties to petroleum-based polymers [47].

### 3.1. Furan-Containing Aliphatic PAs

The use of FDCA as a monomer in the synthesis of furane-aliphatic PAs is not a novel synthetic challenge. Indeed, as early as 1961, Hopff and Krieger described the synthesis of a series of PAs by condensation of linear amines with different heterocyclic dicarboxylic acids, including FDCA. The PAs obtained in these works, unfortunately, decomposed either during the synthesis itself or during processing at high temperatures. Therefore, at that time, the authors concluded that these materials would not have improved performance compared to their TPA-derived (terephthalic-derived PAs) analogues [48,49]. Probably due to this negative conclusion, it took more than 10 years for another work related to FDCA-based PAs to be described. Indeed, in 1974, Heertjes and Kok reported the synthesis of a series of PAs by the condensation of FDCA or, alternatively, the methyl ester (DMFDCA) or the acyl chloride (FDCACl) with C4, C6 or C8 linear aliphatic diamines [50]. For this purpose, two different experimental protocols were employed: interfacial solution and melt polymerization. The main drawback described by these authors was the decarboxylation of the FDCA, which occurred above 195 °C. Indeed, the decarboxylation of the acid was the reason that led them to use the dimethyl ester, which has higher thermal stability, or the acyl chloride derivative, which is more reactive, allowing polycondensation reactions at lower temperatures. In any case, both the yields of the polymerizations and the molecular weights of the obtained polymers were considerably better than those previously described by Hopff and Krieger. Synthesized crystalline PAs could be used to make fibers and films and had melting temperatures (T_m_) between 250 °C and 125 °C. However, it should be noted that these materials still had a long way to go before they could be considered analogous in performance to TPA derivatives. Again, it took a long period of time, until 2009, when O. Grosshardt et al. described the synthesis of PAs by condensation of FDCA and a series of linear aliphatic diamines through a melt polymerization protocol using Sn or Ti derivatives as catalysts. The obtained PAs, in this case all amorphous, presented complete decomposition temperatures between 350 °C and 450 °C and T_g_ between 70 °C and 100 °C [51].

Subsequently, in 2014, Gruter et al. [52,53], in two successive patents, described that when DMFDCA dimethyl ester was used as the starting product and subjected to high temperatures for polycondensation, extensive *N*-methylation of the obtained PA occurred. This undesirable secondary reaction dramatically worsened the thermal and mechanical properties of the materials. To avoid it, a two-step protocol was proposed based on a first stage, with a very strict temperature control, in which FDCA was condensed with an excess of the corresponding diamine. In the second stage, the previously obtained mixture of oligomers was treated with a bifunctional linker to grow the chain.

**Scheme 7:**
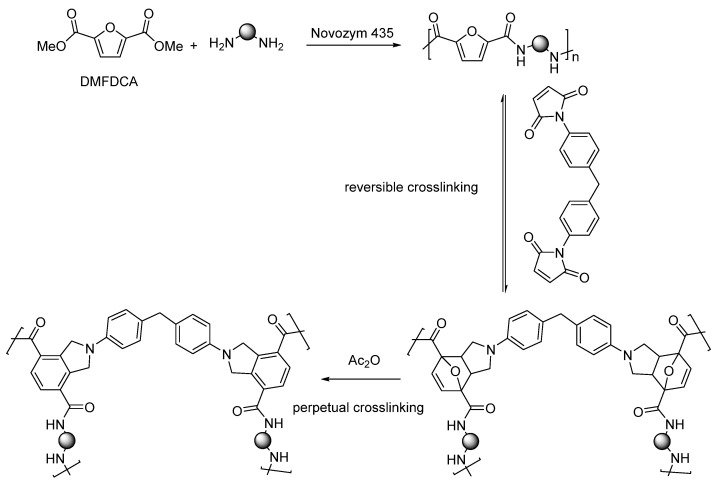
Enzymatic synthesis of a bio-based PA through the reaction between DMFDCA and a series of diamines. Reversible and perpetual crosslinking.

In 2015, Y. Jiang et al. [54] described the first enzymatic synthesis of a furane-based PA employing DMFDCA and 1,8-octanodiamine as starting building blocks. Novozym 435, an immobilized form of *Candida antarctica* Lipase B (CALB) on acrylic resin, was employed as a biocatalyst. The choice of the diester as monomer was based on the higher solubility and lower T_m_ compared to the diacid. Regarding the amine, the better catalytic activity and the higher selectivity of lipases toward long-chain aliphatic amines were taken into account. The obtained molecular weights, up to 54 kDa, were much higher than those previously obtained through melt polymerization, with the great advantage that, in the enzymatic alternative, high temperatures were not required. The so-obtained PA8F resulted in a promising sustainable equivalent to terephthalic acid-derived PA8T with similar T_g_ and thermal stability but with lower T_m_. It is true that this work presented a challenge to improve. Indeed, toluene and diphenyl ether were used as solvents that cannot be considered green alternatives. However, the high T_m_ of the starting diester derivative and the tendency of diamine to sublimate made the solvent-free process unfeasible. Subsequently, these same authors described the reversible cross-linking of these materials through temperature-regulated Diels-Alder reactions between the furanic rings and 1,1′-(methylenedi-4,1-phenylene)bismaleimide. Alternatively, to this reversible crosslinking, aromatization of the materials was shown to provide perpetual crosslinking (see Scheme 7) [55].

In 2017, M. Cao et al. [56] described the synthesis of a new semiaromatic PA condensating terephthalic acid and FDCA with 1,10-diaminodecane through an existing industrialization process based on a first prepolymerization step followed by a solid-state polymerization (SSP) phase. The last step was controlled online by a TG-IR equipment, monitoring the FDCA decarboxylation reaction. The exhaustive analysis of the structure of the obtained PA revealed that, probably due to the aforementioned decarboxylation process, the ratio of the furanic moieties incorporated into the polymeric chain was considerably lower than the feeding ratio of the monomers. The obtained copolymeric PA, PA10T/10F, presented a T_m_ higher than 280 °C and, to the delight of the authors, a stability comparable to the PA10T homopolymer. The authors attributed this excellent stability to the fact that not all the furanic rings incorporated in the polymeric chain had carboxyl groups, the cause of FDCA instability.

**Scheme 8:**
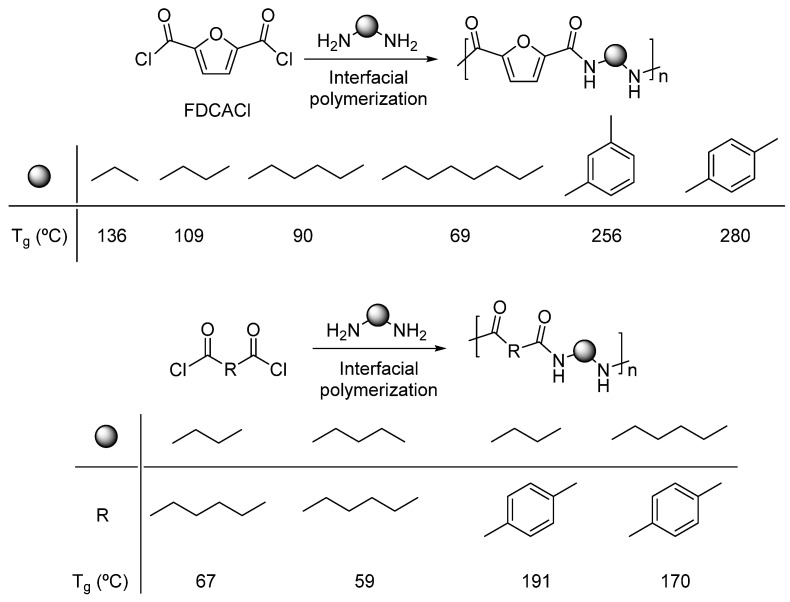
Polycondensation reaction of FDCACl and diverse diamines. Comparison with aliphatic and aromatic non-biobased PAs.

**Figure 4:**
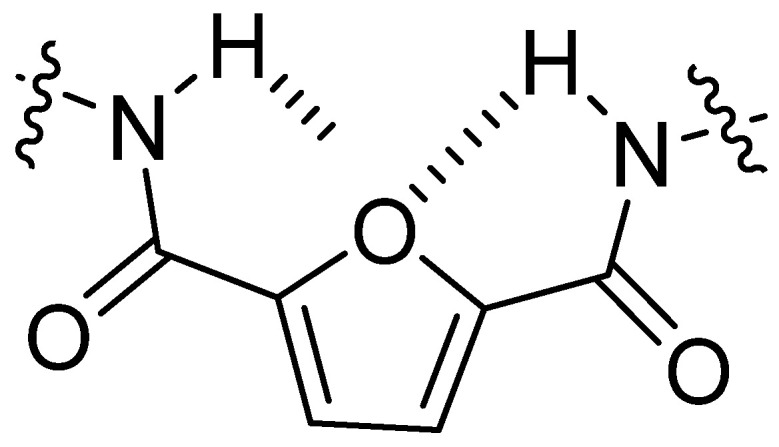
“Double bent” conformation of the furan moiety enabling the formation of intramolecular hydrogen bonds.

In the same year, a comprehensive comparative study was carried out by L. Cureton et al. In this work, a wide variety of PAs were synthesized using an interfacial polymerization protocol. For this purpose, the acyl chloride derivative of FDCA, FDCACl, was condensed with structurally diverse aliphatic and aromatic diamines (Scheme 8). In addition, with comparative intentions, different aliphatic Nylons and semiaromatic PAs were prepared through the same methodology. After properly characterizing the obtained materials by means of GPC, NMR and IR, some of their properties were compared in order to establish structure-property relationships. The first difference between furan-based PA and those lacking this heterocyclic ring was the solubility. Notably, all furane-derived PAs were soluble in polar aprotic solvents such as DMF, DMP or THF, while all Nylons or semiaromatic PAs were completely insoluble in these solvents. This improved solubility of the biobased PAs can facilitate their subsequent processing and expand their range of applications. Analogously, notable differences were detected in the thermal behavior of the polymers. The two fully aliphatic PAs synthesized in this work (Nylon 4,6 and Nylon 6,6) turned out to be crystalline polymers with T_m_ of 262 °C and 225 °C, respectively [57], which is completely consistent with the values previously reported in the literature and with computational predictions [58]. In contrast, the two semiaromatic PAs exhibited very different thermal behavior depending on the aliphatic chain length. Thus, poly(hexamethylene terephthalamide) showed a very significant melting peak around 270 °C, although the degree of crystallinity was lower than in the fully aliphatic PAs. In poly(butylene terephthalamide), however, no crystalline regions were detected. Therefore, it was concluded that the benzene aromatic rings disrupt the crystal packings that remain with considerable aliphatic chain lengths. In contrast, all biobased furan PAs were found to be completely amorphous regardless of the aliphatic chain length. That is, the furan moiety hinders the formation of crystalline areas more effectively than the benzene ring, probably due to the kinked architecture of the furan-based polymeric chains that weakens and even prevents hydrogen bonds between amide groups [57]. Interestingly, Karam and colleagues demonstrated through computational analysis that the double bent conformation, in which both dihedral angles are 0° (see Figure 4), is the most stable. In this conformation, hydrogen bonds between the amidic hydrogen and the furanic oxygen are greatly favored, and, therefore, hydrogen bonds between the amide functions of different chains are minimized. Furthermore, this rigidity, which limits the degrees of freedom of the chains, was also reflected in the T_g_s. Thus, furanic PAs presented the highest T_g_s compared to aliphatic and poly terephthalamides. As expected, due to the major flexibility of the aliphatic segment, the longer the aliphatic chain, the lower the T_g_. This trend was maintained in all three types of PAs [59].

**Scheme 9:**
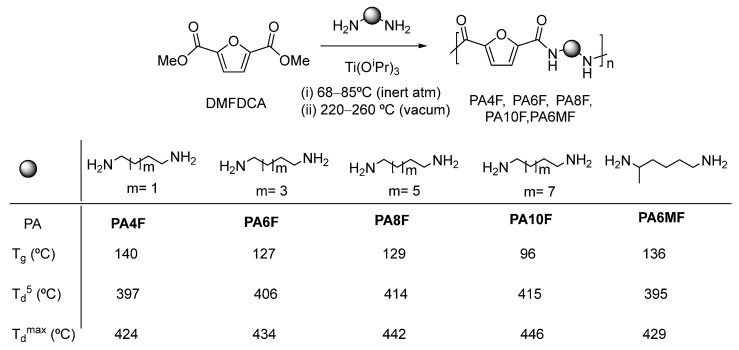
Synthesis of furan-derived PAs via two-step polymerization reaction with a series of aliphatic diamines and low concentrations of titanium isopropoxide.

More recently, M. Kamran et al. described the synthesis of a series of PAs employing DMFDCA and aliphatic amines with different chain lengths. In this case, an environmentally benign protocol was employed based on a melt polycondensation technique and using very low concentrations of titanium isopropoxide (TIPT) as a catalyst (see Scheme 9). The relatively high molecular weights of the synthesized PAs (M_n_ = 8–11 kDa), the amorphous character of most of them and the thermal stability comparable to the fossil-based analogues are the most relevant results of this work. It is worth mentioning that, in addition to the expected amines and esters, other end groups were detected by means of MALDI due to diverse secondary reactions promoted by the high temperatures necessary for the polymerization [60].

**Scheme 10:**
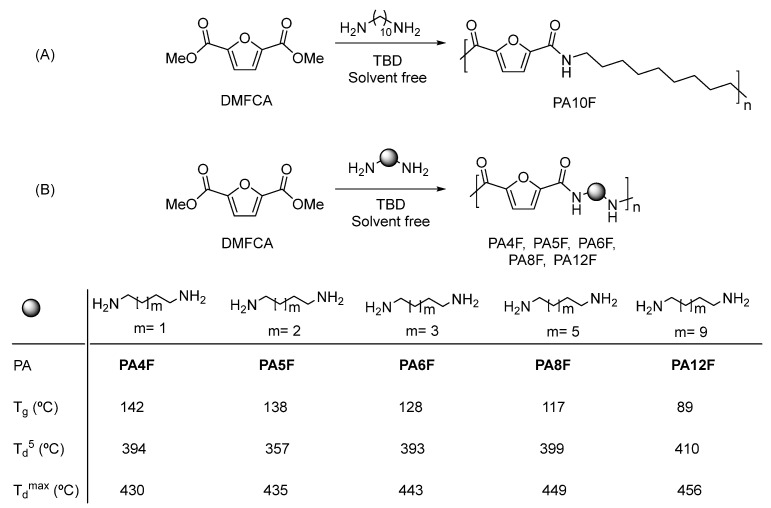
(**A**) Bulk polymerization of DMFCA and 1,10-decanediamine using TBD as a catalyst. (**B**) Extension of the methodology to other aliphatic linear amines and thermal properties of the obtained PA.

In the same year, S. Xie et al. described the polycondensation between the diester derivative DMFDCA and 1,10-decanediamine obtained from castor oil under different reaction conditions. The main difference with the previous work was that, in this case, the bulk polymerization was catalyzed by 1,5,7-triazabicyclo[4.4.0]dec-5-ene (TBD), a versatile superbasic and non-toxic organocatalyst (Scheme 10A) [61]. Furthermore, this paper, together with a later one reported by the same authors, constitutes, as far as we know, the only studies of the mechanical properties of this type of semiaromatic PAs. Notably, for PA10F, these authors described a tensile strength of 52.12 MPa, a tensile modulus of 1636 MPa and an elongation at break of around 69% [62]. Subsequently, they carried out an exhaustive study on the influence of the aliphatic chain length of the diamine monomer on the thermal and mechanical properties of the prepared polymers (Scheme 10B) [63].

**Scheme 11:**
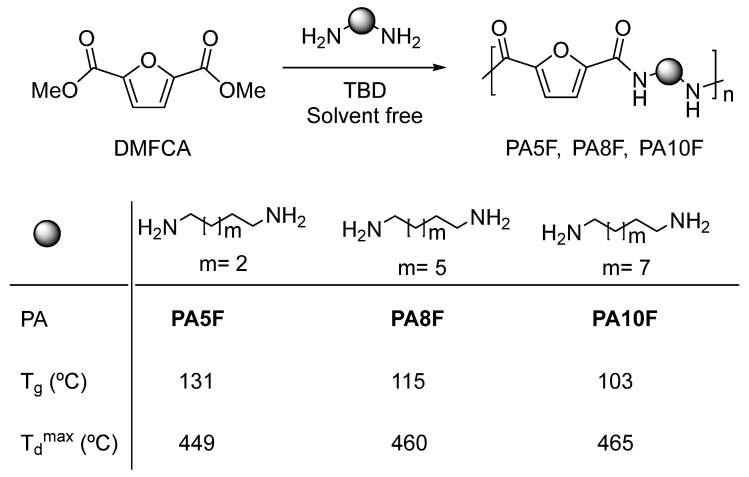
Synthesis of furan-derived PAs using continuous flow technology, and the thermal properties of the synthesized PAs (PA5F, PA8F and PA10F).

The same diester was employed as a monomer by Y. Feng et al. in 2024 [64]. In this case, an elegant and eco-friendly continuous flow technology was used to carry out the polycondensation between the furane-based diester monomer and a series of linear aliphatic diamines, using TBD as a catalyst. At elevated temperatures, methanol by-product vaporizes, creating gas-liquid slug flow in the microchannel and sharply reducing reactant residence time, from hours to minutes, leading to lower polymer yield and molecular weight. This insight suggests that a variable-temperature two-stage polymerization could improve efficiency. Besides, the mild conditions employed prevented secondary reactions such as *N*-methylation (Scheme 11). Moreover, in addition to the synthesis and the characterization of the PAs, these authors described the chemical depolymerization of the materials to obtain the monomeric precursors, which is extremely important from an environmental point of view. Indeed, chemical degradation opens the way to chemical recycling, a better alternative than the commonly carried out mechanical recycling, as the raw monomers obtained can be reused to synthesize virgin polymers again.

**Scheme 12:**
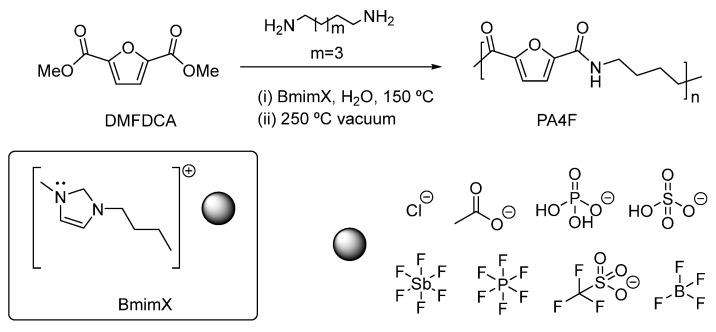
Synthesis of aliphatic PA4F via a two-step polymerization process using 1-butyl-3-methylimidazolium (Bmim) derivatives as catalyst.

At this point, it is important to mention the pioneering work recently published by J. Feng et al., which represents an important step towards more sustainable protocols (Scheme 12). These authors proposed the replacement of metallic or superbasic derivatives with liquid ions as catalysts. Notably, PA4F was efficiently synthesized in a two-step methodology. In the first stage, the prepolymerization took place employing different ionic liquids, all of them based on 1-butyl-3-methylimidazolium (Bmim) as catalyst and water, the greenest alternative, as solvent. In the second step, the melt polycondensation of the oligomers was carried out. The so obtained PAs showed satisfactory M_n_ (up to 27 kDa), elevated T_g_ (around 150 °C) and excellent elastic modulus (3.7 GPa) [65].

**Scheme 13:**
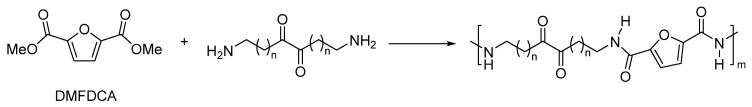
Polycondensation of DMFCA with long-chain aliphatic diamines containing oxa-lamide groups.

It has already been mentioned in this section that, due to the structure of the furan ring, hydrogen bonding between neighboring chains is hindered in favor of the corresponding intramolecular interactions. This fact influences the crystallinity of the materials and, consequently, their thermal and mechanical properties. Recently, Zhan et al. synthesized a series of PAs by melt polycondensation of DMFDCA with long-chain aliphatic diamines containing oxalamide groups. Their strategy was to minimize intramolecular interactions by forming double hydrogen bonds between the oxalamide moieties of adjacent chains. Indeed, the crystallinity of the obtained materials increased and their properties were improved. Furthermore, the excellent chemical degradability of these PAs was demonstrated. In fact, when they were subjected to basic hydrolysis conditions, the starting monomers were recovered with excellent conversions (Scheme 13) [66].

Furan-based PAs have generally emerged as promising bio-based alternatives to petroleum-derived materials, offering good thermal stability, high glass transition temperatures, and notably improved solubility, which significantly facilitates processing. However, a major challenge lies in the variability of their properties, which complicates the predictability of performance. In fact, some properties show considerable variation between studies, even for structurally similar compounds, as experimental synthesis conditions can have a substantial impact on the final material characteristics.

Table 1 compiles all the works discussed in this section, emphasizing the most relevant aspects of each.

### 3.2. Furan Containing Aromatic PAs

To the best of our knowledge, the first report regarding the synthesis of furan-aromatic PAs dates back to 1964. In this pioneering work, the synthesis of a series of PAs from FDCACl and various aromatic diamines was described. Unfortunately, the characterization of the materials was not very detailed. Indeed, only the melting points, degradation temperatures, and inherent viscosities of some of them were given [67].

Later, Mitiakoudis and coworkers described the synthesis of an extensive series of furane-aromatic and all furane PAs. In this case, not only FDCA and the corresponding chloride, but also the 3,4-disubstituted regioisomers were used. After exhaustive optimization of the polymerization conditions in terms of yields and molecular weights of the obtained materials, these were characterized, and some of their properties were compared. One of the main conclusions obtained by these authors was that furane-aromatic PAs presented, in general, better properties than wholly furane PAs [68,69].

**Scheme 14:**
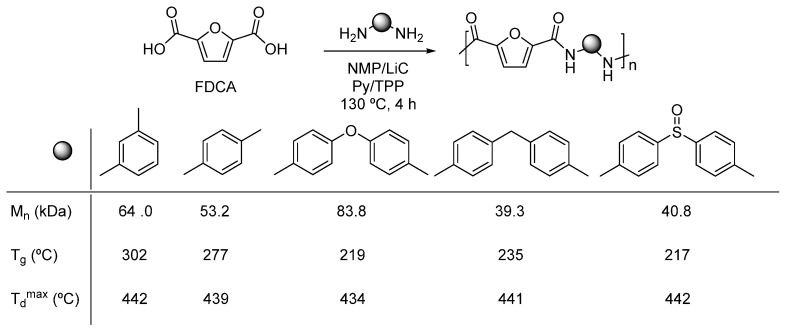
Direct polycondensation in NMP and TPP between FDCA and a series of aromatic diamines.

Luo and coworkers described the direct polycondensation between FDCA and a series of aromatic diamines (Scheme 14). The so-obtained partial biobased materials were properly characterized by means of FT-IR, NMR and GPC. In addition, the mechanical properties of the prepared polymers were also analyzed, and the influence of the structure of the diamine monomer on them was studied. Therefore, all the synthesized PAs presented adequate mechanical properties, highlighting the results obtained with the PA synthesized with *para*-phenylenediamine, presumably due to the rigidity of the polymeric skeleton and the high molecular weight. On the other hand, all of the PAs prepared in this work were completely soluble in common organic solvents, which can greatly facilitate processing compared to insoluble fossil-derived aromatic counterparts [70].

**Scheme 15:**
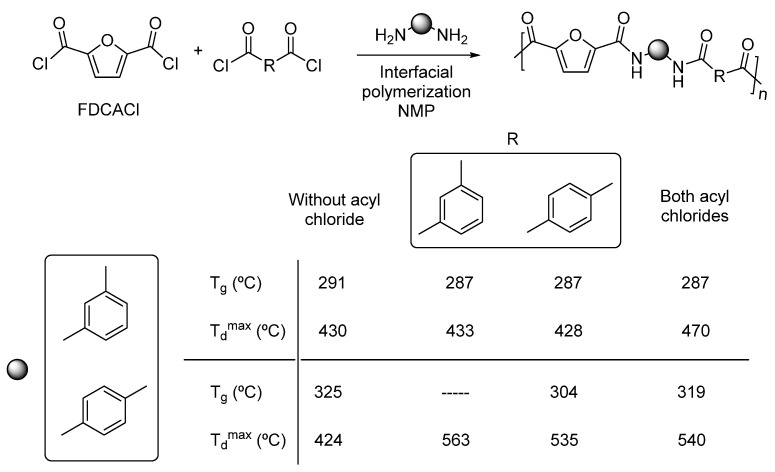
Synthesis of furane containing full aromatic PAs starting from FDCACl, *meta* and *para*-acyl chloride isomers and *meta* and *para*-diamine isomers.

In 2020, Yu and coworkers reported the synthesis of an extensive series of furane-containing full aromatic PAs through melt polycondensation reaction at low temperatures. The employed substrates were FDCACl, *m*-phthaloyl chloride (MPC), and terephthaloyl chloride (TCL) and *m*-phenylenediamine (MPD), *p*-phenylenediamine (PPD), as diamine monomers (Scheme 15) and different combinations between them were performed, obtaining eight furane-containing PAs. An exhaustive characterization of all materials was accomplished by means of NMR, XRD, DSC and TGA. In addition, the solubility of the obtained polymers in different organic solvents was tested. The principal conclusion found in this work was that the furane-containing materials presented similar thermal behavior and thermal stability to those of the traditional aromatic PAs. Regarding the solubility of these materials, these authors drew the same conclusion previously described by Luo et al.; in fact, these biobased PAs presented the great advantage of being soluble in some organic solvents, which really facilitates their processing [71]. 

**Scheme 16:**
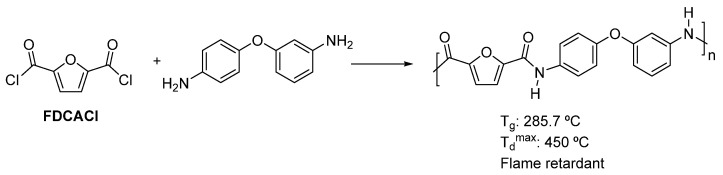
Polycondensation reaction between **FDCACl** and 3,4′-diaminophenylether.

The same methodology was used a few months later by Cao and coworkers, thus, a polycondensation at low temperature between FDCACl with the biobased 3,4’-diaminophenylether (Scheme 16). The so-obtained polymer showed excellent solubility and spinnability, and a fiber with a smooth surface and uniform thickness was fabricated, which, in addition, exhibited outstanding thermal and mechanical properties and flame-retardant quality [72].

**Scheme 17:**
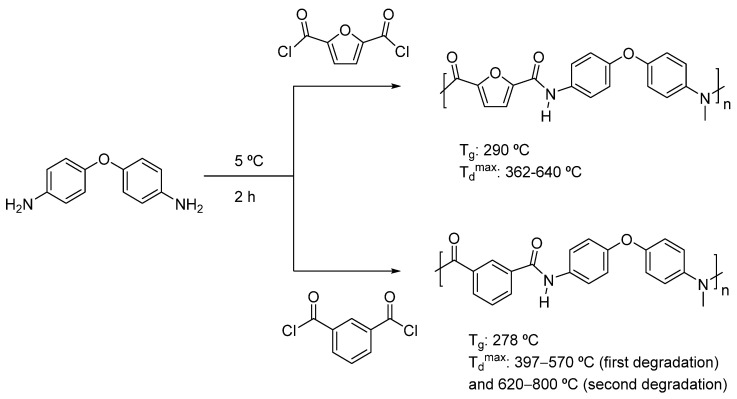
Polycondensation reaction between 4,4′-diaminodiphenyl ether and FDCACl. Comparison between furan-derived and isophthalic-derived PA thermal properties.

More recently, the 4,4’-regioisomer of the diaminophenyl ether was subjected to the polycondensation reaction with FDCACl (Scheme 17). The so-obtained biobased PA was properly characterized and compared to the isophthalic-derived PA, which presents a fossil origin. The furane-containing polymer displayed analogous or even better thermal, mechanical and barrier properties. Moreover, the hydrolysis of the polymeric materials was performed under basic conditions and the starting monomers were recovered. Molecular dynamic simulations revealed that the presence of the heterocyclic ring lowered the energy barrier of the hydrolysis process [73]. Therefore, the furan ring not only increases the biogenic content of the materials but could also facilitate their chemical recycling, which is extremely important from an environmental perspective.

Table 2 summarizes the studies discussed in this section, presenting the key data and the structures of the materials synthesized in each case.

### 3.3. Multifuran Monomers

**Scheme 18:**
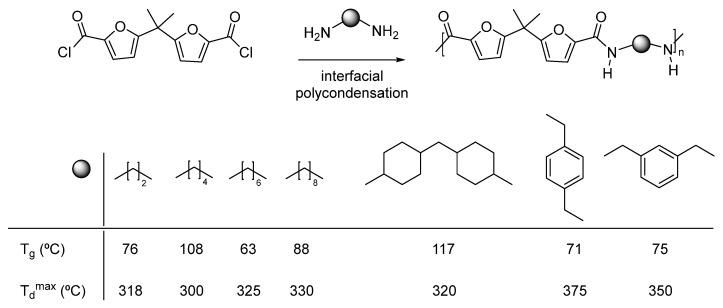
Interfacial polycondensation between 2,2′-*bis*[2-(5-chloroformyl)furfuryl]propane and a number of structurally diverse diamines.

In addition to FDCA and its immediate derivatives like the dimethyl ester and diacyl chloride, other monomers that incorporate the furan ring into the polymer backbone have been developed. Indeed, encouraged by the promising properties that the furan heterocycle imparts to materials, different polymers have been prepared using monomers with two furan rings connected by different linkers. In this sense, Gharbi et al. synthesized a series of PAs containing a bifuranic structure, condensating 2,2′-*bis*[2-(5-chloroformyl)furfuryl]propane with a number of structurally diverse aliphatic, cycloaliphatic or benzylic diamines (Scheme 18). A comprehensive study on the influence of the diamine structure on both the process yields and the thermal properties of the obtained material was conducted in this contribution [74].

**Scheme 19:**
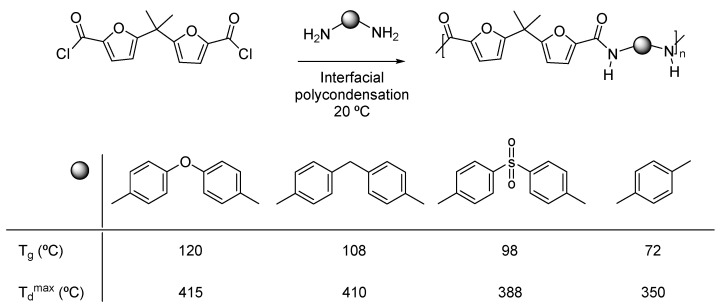
Interfacial polycondensation between 2,2′-*bis*[2-(5-chloroformyl)furfuryl]propane and aromatic diamines.

Later, this protocol was extrapolated to aromatic diamine monomers with the principal objective of improving the properties of the materials due to the stiffness provided by the aromatic rings (Scheme 19) [75].

**Scheme 20:**
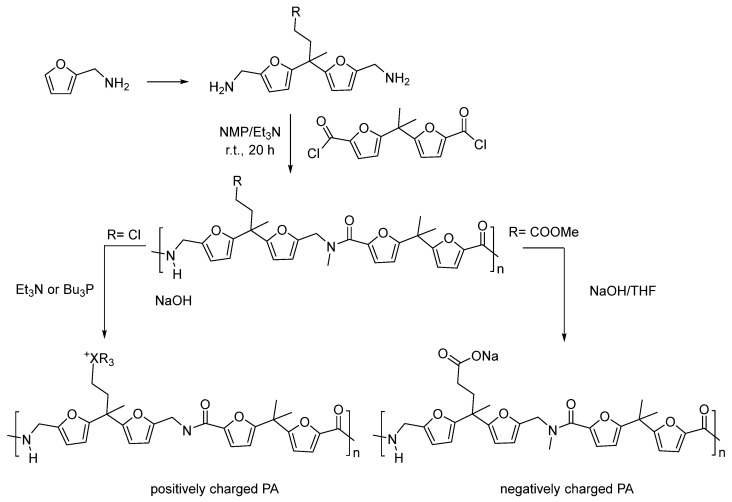
Synthesis of a series of bisfuran-based PA with different spacer moieties employing furfuryl amine as a starting material. Post-polymerization reaction obtains positively and negatively charged PAs.

Interestingly, this type of monomer allows the incorporation of different functional groups into the spacer that joins the two furan rings, which also allows the modulation of the polymer properties. In this sense, recently, Shu and collaborators have described the synthesis of a series of bisfuran-based PAs with different spacer moieties employing furfuryl amine as starting material (Scheme 20). Moreover, in addition to studying the influence of the spacer on the thermal properties, crystallinity, solubility or water absorption, the obtained PAs were subjected to different post-polymerization modification processes, achieving positive and negatively charged functionalized bio-PAs [76]. Furthermore, these authors have demonstrated the high capacity of these cationic PAs as polyfluoroalkyl (PFA) environmental contaminants adsorbents [77].

**Scheme 21:**
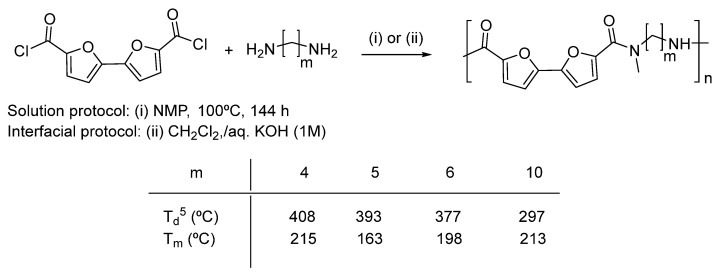
Solution and interfacial polycondensation of bifuran-derived dicarbonylchloride with several aliphatic diamines.

More recently, the bifuran moiety, in which two furan rings are directly linked through the 2-positions, has received more attention. In this structure, the π conjugation is extended mainly due to the complete coplanarity of the rings and the dihedral angle between them of 180°. Moreover, the rotational barrier between the heterocycles is relatively high, which confers superior rigidity to this bifuran-derived building block compared to those derived from the biphenyl moiety. Taking into account these structural characteristics and the possibility of synthesizing them from biobased furfural [78], different monomers with a bifuran structure have been used in the synthesis of polymeric materials, with the hypothesis of improving the thermal and mechanical properties, and, in consequence, the performance of the obtained materials [79,80]. In this context, regarding PAs, N. Miyawaka et al. described the polycondensation of bifuran-derived dicarbonylchloride (see Scheme 21) with several aliphatic diamines through solution and interfacial methodologies. Indeed, as expected, the prepared PAs exhibited higher T_m_ compared to the analogous PAs bearing a single furan ring. Moreover, PAs containing the bifuran structure also turned out to be more thermally stable [81].

**Scheme 22:**
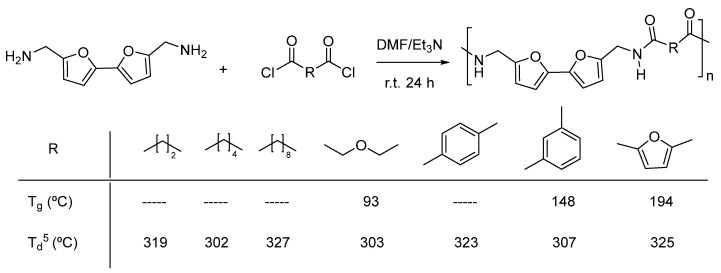
Polymerization of bifurfurylamine and diacyl chlorides with different chemical structures via solution polycondensation.

In contrast, K. Arai et al. reported the condensation of bifurfurylamine (see Scheme 22), prepared starting from biomass-derived furfurylamine, with seven structurally different diacyl chlorides. Newly, in this work, the superiority of polymers carrying the bifuran moiety compared to those presenting a single furan ring was demonstrated [9].

In summary, bifuran-based monomers have attracted interest due to their extended π-conjugation, planarity, and high rotational barrier, which confer greater rigidity compared to single-furan analogues, demonstrating enhanced thermal performance for bifuran-based PAs. Moreover, recent studies have explored the incorporation of functional groups into the spacer, expanding structural diversity and property control. However, while promising, the structural complexity and synthetic accessibility of such monomers warrant further investigation to assess their scalability and practical advantages over simpler furan-based systems. Table 3 provides a summary of the works analyzed in this section, highlighting the most relevant data and the structure of the materials synthesized in each study.

## 4. Terpene Derived PAs

Terpenes constitute a group of organic natural molecules, generally secondary metabolites, that exhibit remarkable functional and structural diversity. For example, some terpenes serve as defense agents in insects [82], while others act as growth regulators [83], among various other biological roles. Regarding the chemical structure, all terpenes share the common characteristic of the repetition of C5 isoprene units [6,84], although their architectures can vary. Indeed, terpenes can be acyclic, mono, di or even polycyclic. Additionally, the number of repeating isoprenes is another criterion for classifying terpenes: monoterpenes (C10), sesquiterpenes (C15), diterpenes (C20), sesterterpenes (C25), triterpenes (C30) and so on [85]. Most terpenes are easily extracted from non-edible plant parts, and some of them are extremely abundant in nature. Therefore, their abundance and structural diversity make them excellent natural, economically feasible building blocks with the potential to replace fossil sources as raw materials in polymer synthesis [13].

**Scheme 23:**
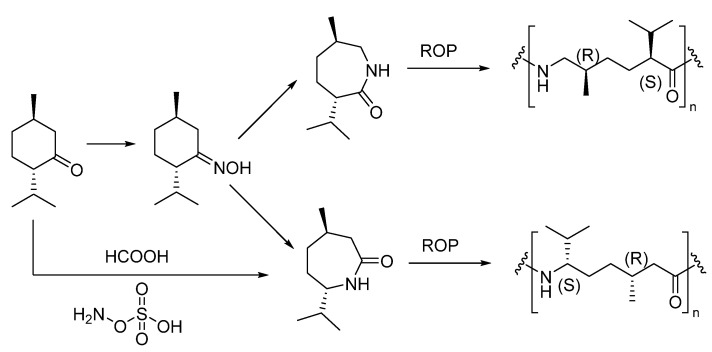
Synthesis of lactams starting from menthone and posterior ROP to synthesize PA oligomers.

As previously discussed, the ROP of ε-caprolactam is the conventional methodology for the synthesis of Nylon 6 [85]. Similarly, the vast majority of terpene-based PAs are synthesized by ROP from the corresponding lactams. Indeed, the most widely used methodology for obtaining the lactam monomer is the Beckmann rearrangement from the oxime, which is usually prepared from the ketone derivative. In this context, Rieger and Winnacker proposed the use of terpenoid ketones, like menthone, for the synthesis of biobased PAs. For this purpose, two alkyl-substituted lactams regioisomers were prepared via Beckmann rearrangement and subsequently polymerized through a ROP protocol in both acid and neutral conditions (Scheme 23). These initial studies laid the groundwork for the investigation of terpene-derived PAs with very interesting structures carrying pendant groups and stereocenters in the backbones, enabling fine-tuning of key properties such as thermal behavior, crystallinity, solubility, and mechanical performance. Additionally, it should be mentioned that in this work, it was demonstrated that the tacticity of the synthesized polymers was dictated by the stereochemistry of the monomers [86].

Certainly, most terpene-based PAs have been prepared following an analogous synthetic pathway. Table 4 summarizes the most representative works on the synthesis of PAs using terpenes as starting substrates, focusing on the structure of the materials obtained and the most relevant aspects. In this context, recently, Stockmann et al. demonstrated that limonene oxide is a suitable monomer for the synthesis of PAs with two stereocenters per repeating unit and a pendant functionalizable isopropylene moiety (Entry 2). Rewarding the thermal properties of the obtained materials, the absence of melting points suggested a high amorphous character with T_g_s up to 120 °C [13].

**Scheme 24:**
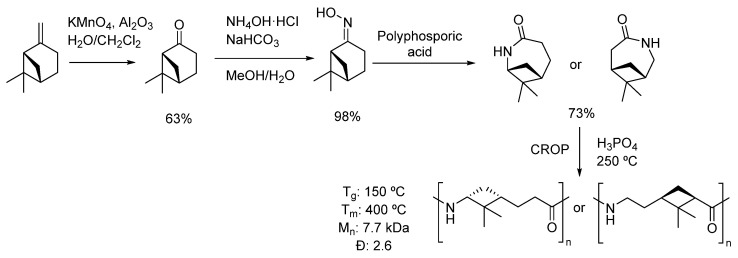
Synthesis of β-pinene-derived PAs, converting β-pinene into oximes and then into lactams, which are then converted into PAs through cationic (using H_3_PO_4_, HCl and aminocaproic acid at 250 °C) ROP.

Similarly, α- and β-pinene regioisomers have been used in the synthesis of PA employing both cationic and anionic protocols in the ROP reaction (Entries 3–5). For instance, Winnacker et al. described the synthesis of the stereo-regular PAs starting from β-pinene-based monomer (Scheme 24), with excellent thermal properties with T_m_ of around 400 °C and T_g_ about 150 °C [87]. In this case, again, the stereoinformation of the monomer was transferred to the polymer chain. Moreover, a regioisomer lactam was obtained in the Beckmann rearrangement from the corresponding oxime. This secondary product was also submitted to polymerization conditions, successfully obtaining an alternative PA. However, considering that the starting substrate was obtained in very small quantities, this PA could not be characterized so exhaustively.

Carene, a cyclic terpene found in the essential oils of various plants, has also been employed in the synthesis of PAs through β- and ε-lactam intermediates. For instance, in 2019, Sieber and co-workers [88] achieved α-pinene and (+)-3-carene-derived PAs. The β-lactams were synthesized by [2+2] cycloaddition of chlorosulfonyl isocyanate to either α-pinene or (+)-carene. Then, these lactams were polymerized through the anionic ROP using NaH or potassium as initiators and benzoyl chloride (BzCl) or acetic anhydride (Ac_2_O) as pre-activators. Among the obtained polymers, synthesized from (+)-3-carene, there is particular interest because of the molecular weight exceeding 30 kDa. Besides, the high thermal stability of the synthesized PAs, DSC analysis also noticed the absence of T_m_, which indicates a highly amorphous nature, advantageous for applications in optics and electronics (Entries 4–**5**) [89].

To finish this section, in 2024 Kleybolte and Winnacker presented simple anionic ring-opening polymerizations (AROP) of β-pinene lactam in bulk and in solution, and they made the proof of different initiators, all of them green alternatives, with the aim of using these polymers in biomedical applications. Thereby, concluded that NaH is the most successful for in-bulk polymerization with molecular weights of 28.9 kDa, and for solution-AROP, *i*PrMgCl·LiCl, obtaining polymers with high molecular weights (M_n_ = 9.4 kDa). Their good mechanical, thermal (T_g_s up to 195 °C and T_d_s up to 440 °C), and transparent appearance render them promising high-performance biomaterials [90]. 

**Table 4:** Synthesis of terpene-based PAs through ROP reaction.

Entry	Substrate	ROP	Properties of PAs	Structure	Ref.
**1**	Limonene oxide	Anionic	▪ Amorphous ▪ T_g_ = 120 °C	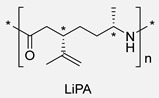	[13]
**2**	β-pinene	Cationic	▪ T_m_ = 400 °C ▪ T_g_ = 150 °C	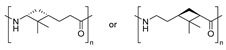	[87]
**3**	β-pinene	Anionic	▪ T_m_ = 308–322 °C ▪ T_g_ = 150 °C	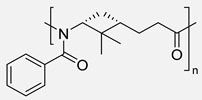	[89]
**4**	α-pinene	Anionic	▪ Amorphous ▪ T_g_ = 120 °C	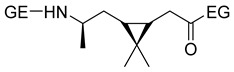	[88]
**5**	(+)-3-carene	Anionic	▪ M_n_ = 30 kDa ▪ Amorphous	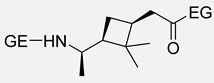	[88]
**6**	β-pinene	Anionic	▪ M_n_ = 28.9 kDa in bulk and 9.4 kDa in solution. ▪ T_d_ > 440 °C ▪ T_g_ > 195 °C	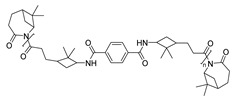	[90]

**Scheme 25:**
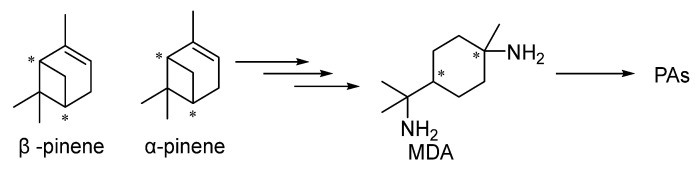
Synthesis of PAs starting from α-pinene and β-pinene compounds, which are reacted to obtain the monomer MDA.

As it has been mentioned, ROP is by far the most widely used methodology in the synthesis of PAs from terpene or terpenoid substrates. However, a few examples of access to terpene-based PA via polycondensation processes can also be found in the literature. Certainly, the presence of two double bonds in the limonene structure allows easy access from this cyclic monoterpene to difunctionalized monomers through epoxidation or hydration reactions, among others [91]. One of the pioneering authors in this context was Trumbo in 1988, who employed 1,8-diamino-*p*-menthane (MDA), a readily available limonene derivative, to synthesize PAs via interfacial condensation with various diacid chlorides (Scheme 25) [92]. This study presented MDA as a renewable alternative to petroleum-based diamines in polymer chemistry. Although the polymers obtained in this pioneering work did not present satisfactory molecular weights, it laid the foundation for subsequent research in this area.

**Scheme 26:**
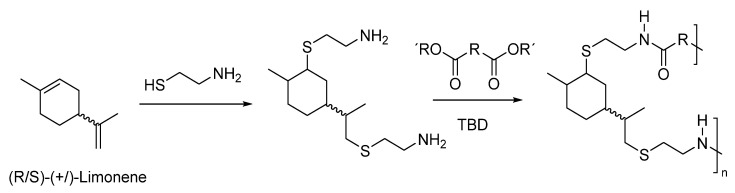
Limonene-derived PA synthesis via polycondensation between limonene-derived diamine monomer and a series of diesters.

Some years later, in 2013, Firdaus and Meyer carried out a similar work using this terpene in the synthesis of various polymers, including PAs (Scheme 26). The reactivity of the double bonds presented in the racemic limonene and the successful thiol-ene *click* reaction was an elegant combination for the access to difunctional monomers. Indeed, the obtained enantiomerically pure limonene-derived diamine monomers were reacted with various biobased diesters employing TBD as a catalyst, obtaining renewable PAs with molecular weights up to 12 kDa. Additionally, they observed that the presence of cycloaliphatic bulky groups prevented intermolecular interaction, leading to amorphous PAs with T_g_ values around 40 °C. The study revealed that the chirality of the employed monomers did not affect the thermal properties of the PAs [93].

As a conclusion, we can say that terpene-derived PAs offer clear advantages such as structural diversity, thermal stability, and the potential for property tuning via pendant groups and stereocenters. Their synthesis via ROP is well-established and enables access to high-performance, biobased materials. However, challenges remain, including low molecular weights in some cases, limited crystallinity, and potential scalability issues. The influence of stereochemistry on bulk properties is not always significant, warranting further study.

## 5. Fatty Acid-Derived PAs

**Scheme 27:**
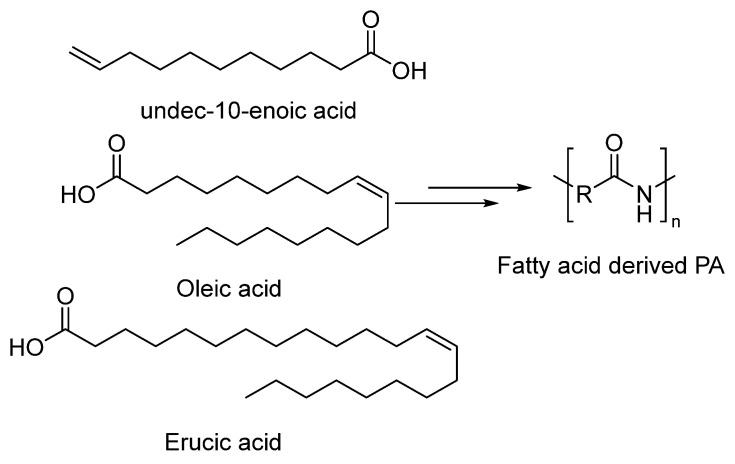
Chemical structure of undec-10-enoic, oleic and erucic acids, suitable candidates for the synthesis of biobased PAs.

Oils and fats of vegetable and animal origin have historically been, and continue to be, among the most important renewable feedstocks for the chemical industry. Among fatty acids, ricinoleic acid (from castor oil) [94,95], linoleic acid and oleic acid (from sunflower oil) and palmitic acid (from palm oil) are particularly notable as raw materials for chemical production. Fatty acids possess long aliphatic chains and functional carboxylic acid groups, making them excellent candidates for the development of bio-based monomers. Their structural versatility allows for a wide range of chemical modifications, enabling the synthesis of diamines, diacids, and other intermediates suitable for polycondensation reactions [17]. In particular, undec-10-enoic acid, oleic acid, and erucic acid stand out as ideal precursors for step-growth monomer synthesis for PA due to their bifunctional nature and high availability (Scheme 27) [94]. Thus, these three fatty acids can be regarded as key starting materials for sustainable polymer chemistry and will be the primary focus among fatty acids in this review.

**Scheme 28:**
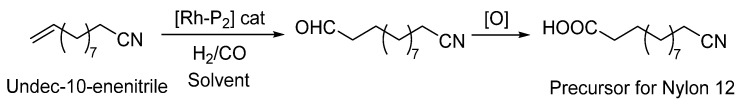
Hydroformylation and later oxidation of undec-10-enenitrile to synthesize PA12.

An example of the use of undec-10-enoic acid derivatives was reported by J. F. Carpentier and coworkers. In this study, undec-10-enenitrile was hydroformylated using the catalyst (dicarbonyl)rhodiumacetoacetonate-biphephos [Rh(acac)(CO)_2_-biphephos] to selectively produce the linear aldehyde. This intermediate was subsequently auto-oxidized upon exposure to air to yield the corresponding fatty acid, a precursor for nylon-12 (Scheme 28). Notably, this approach minimized by-products/waste formation, in part due to the effective recyclability of the rhodium catalyst. Indeed, the recyclability of the catalyst over 4–5 cycles represents a significant step toward minimizing waste and improving process sustainability. The process requires no additional oxidants, reducing environmental load. However, energy inputs related to pressurized gas handling and metal catalyst preparation still pose challenges for scale-up [96].

**Scheme 29:**
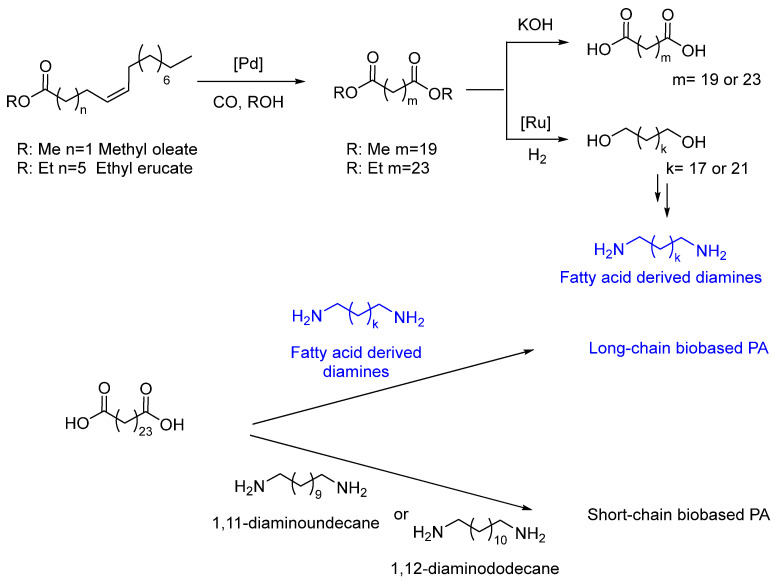
Synthesis of long-chain PAs starting from unsaturated fatty acids, such as methyl oleate and ethyl erucate.

Another example of the use of fatty acids for polymer synthesis was reported by S. Mecking and collaborators. In this case, oleic and erucic acids alkyl esters were used as renewable precursors for the synthesis of biobased polyesters (PEs) and PAs (Scheme 29). For this purpose, methyl oleate and ethyl erucate were firstly subjected to alkoxycarbonylation, and the so obtained diesters were subsequently converted via two parallel routes: (I) saponification to obtain the corresponding dicarboxylic acids, or, alternatively, (II) catalytic hydrogenation using a Ru-based catalyst to afford the corresponding diols. The obtained dicarboxylic acids were condensed with the synthesized long-chain diols, as well as shorter diols, in order to produce tunable chain length PEs. Complementarily, the obtained diols were converted into diamines, specifically, 19-nonadecanediamine and 1,23-tricosanediamine, which were further condensed with the previously synthesized dicarboxylic acids to yield long-chain PAs. Additionally, 1,11-diaminoundecane or 1,12-diaminododecane was reacted with 1,23-tricosanedicarboxylic acid to produce shorter-chain PAs. The obtained PAs exhibited molecular weights of 10 kDa and melting temperatures from 152 °C to 168 °C. As expected, the melting and crystallization temperatures tended to decrease with increasing monomer chain length, indicating that PAs with shorter aliphatic segments possess higher thermal transition temperatures [97].

**Scheme 30:**
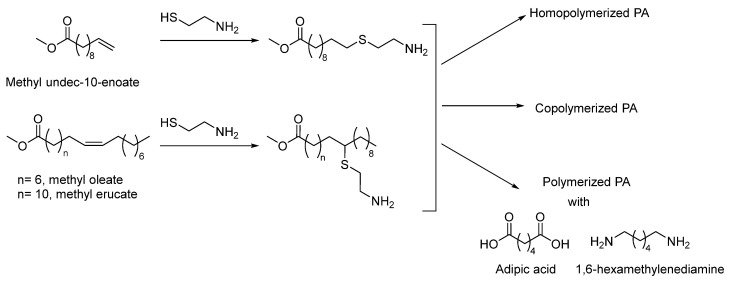
Synthesis of biobased homo and co-PAs starting from undec-10-enoate, methyl oleate and methyl erucate.

Similarly, A. R. Meier et al. employed methyl undec-10-enoate, methyl oleate and methyl erucate as precursors for the synthesis of homo-and co-PAs. In their approach, the double bonds present in these fatty acid esters were functionalized via thiol-ene reaction using cysteamine to produce amino-functionalized monomers (Scheme 30). These monomers were subsequently homopolymerized, copolymerized with each other and polymerized with adipic acid and 1,6-hexamethylenediamine by using TBD as an environmentally friendly, low-toxicity and effective catalyst. The resulting renewable PAs exhibited molecular weights ranging from 3.7 kDa to 15.9 kDa. Additionally, DSC analysis revealed distinct thermal behaviors. Homopolymers derived from undecenoate and erucate derivatives exhibited clear thermal transitions, with T_m_s of 138 °C and 43 °C, respectively. In contrast, the homopolymer of the oleate derivative did not show a detectable melting point, suggesting an amorphous nature. To modulate the broad melting range observed in these homopolymers, copolymerization of monomers was performed. Specifically, undecenoate and erucate monomers were copolymerized, obtaining intermediate melting temperatures relative to their respective homopolymers. This highlights the tunability of the thermal properties of these materials through the appropriate monomer selection. Furthermore, copolymerization of undecenoate monomer with adipic acid and 1,6-hexamethylenediamine, key components of Nylon 6,6, showed that the melting temperatures of the resulting PAs could be finely modulated over a wide range. Notably, the melting points of the copolymers varied from 121 °C to 182 °C, or, in some cases, no melting temperature was observed. For instance, when the molar ratio of adipic acid, 1,6-hexamethylenediamine and undecenoate monomer was 1:1:2, the resulting polymer exhibited no melting point, indicating a highly disrupted crystalline structure. Moreover, increasing the ratio of adipic acid and 1,6-hexamethyeleiamine relative to the undecenoate monomer led to higher melting temperatures. This trend suggests the formation of shorter-chain PAs with an increased proportion of Nylon 6,6-type segments, which contribute to enhanced crystallinity and thermal stability. Collectively, these findings indicated that selective monomer combinations enable the synthesis of PAs ranging from amorphous to high-melting semi-crystalline materials, offering a versatile platform for thermal property engineering [17].

**Scheme 31:**
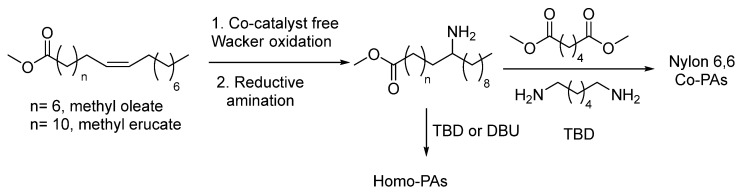
Co-catalyst-free Wacker oxidation of unsaturated FAMEs, subsequent reductive amination to prepare amino FAMEs and polymerization of the synthesized monomers with TBD.

Regarding green approaches, Meier et al. designed an efficient and environmentally benign strategy for the oxyfunctionalization of fatty acid methyl esters (FAMEs) employing molecular oxygen as an oxidizing agent. For this purpose, they subjected methyl oleate and methyl erucate to a co-catalyst-free Wacker oxidation procedure in a dimethylacetamide/palladium(II)chloride solvent-catalyst system. This route allowed for the generation of unsaturated keto-fatty acid esters using molecular oxygen, representing a significant green chemistry advancement. The obtained unsaturated keto-fatty acid esters were further functionalized with amines via reductive amination. The prepared renewable monomers were polymerized with 1,6-hexamethylendimethylamine and dimethyl adipate in order to obtain the corresponding PAs (Scheme 31). The synthesized co-PAs exhibited M_n_ ranging from 11.2 kDa to 21.4 kDa, with dispersities between 1.45 and 2.28. DSC analysis revealed melting points between 224 °C and 250 °C, alongside thermal stability up to 350 °C. Notably, similar to their previous findings, increasing ratios of adipate and 1,6-hexamethyelendiamine resulted in higher melting temperatures. More specifically, increasing the content of amine-based renewable monomers led to a reduction in melting temperatures, attributed to the steric hindrance introduced by the long aliphatic side chains. These results further underscore the tunability of thermal properties in copolymerized Nylon 6,6 PAs. Moreover, the approach highlights another greener synthetic route for fatty acid-derived monomers, benefiting from low catalyst loadings, simplified product isolation, and effective recycling of solvent-catalyst systems [98].

Similar to their previous work, years later, the same authors introduced another innovative procedure to synthesize new dimer FAME monomers using methyl undec-10-enoate, methyl oleate, and methyl erucate as precursors. They employed again the Wacker oxidation to obtain the keto-FAMEs, which were further submitted to reductive amination using aliphatic, cyclic or aromatic diamines in order to obtain partial renewable dimer-monomers, and they even employed amino-FAME in order to obtain fully renewable dimer-monomers. Interestingly, these diamines led to dimer FAME monomers, which were further polycondensed with 1,10-diaminodecane using TBD as a catalyst. The obtained PAs resulted in molecular weights ranging from 24.4 kDa to 33.5 kDa and melting temperatures from 70 °C to 161 °C. Again, they observed that longer aliphatic chains in the precursors led to decreased melting temperatures. Overall, they developed an efficient synthesis strategy to prepare partially and fully renewable dimer fatty acid methyl esters (DFA) from mono-unsaturated fatty acids via catalytic oxyfunctionalization and reductive amination [99].

**Scheme 32:**
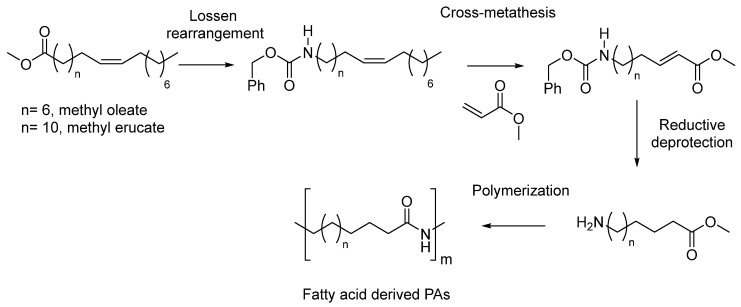
Synthesis of the monomers and fatty acid-derived PAs starting from methyl oleate and methyl erucate.

On the other hand, olefin metathesis has been shown to be a remarkably effective method for the synthesis of fatty acids derived monomers (Scheme 32). In this context, the same FAMEs as in previous works were protected via catalytic Lossen rearrangement. The resulting carbamates were subsequently subjected to cross-metathesis with methyl acrylate. Then, under mild hydrogenation conditions, the remaining carbamate and double bond were hydrogenated, leading to amino-end-functionalized unsaturated FAMEs. These amino-end-terminated FAMEs were further homopolymerized to yield the corresponding PAs. They obtained three PAs exhibiting M_n_ ranging from 14.9 kDa to 22.6 kDa and disparities between 1.73 and 2.20. DSC analysis revealed melting points between 169 °C and 186 °C, with shorter aliphatic PAs exhibiting higher melting temperatures. These thermal properties are comparable not only to those of commercial PAs but also to the previously reported values in this review for similar materials. In this case, the study also explored the mechanical properties of the three obtained PAs. The homopolymerized PAs exhibited Young’s modulus ranging from 1480 MPa to 2320 MPa, indicating that longer aliphatic chains exhibited lower Young’s modulus. This was attributed to a reduced frequency of amide groups and, consequently, diminished hydrogen bonding interactions. Overall, the study demonstrated the potential use of the cross-metathesis strategy for the preparation of new bio-sourced PAs with good properties [100].

**Scheme 33:**
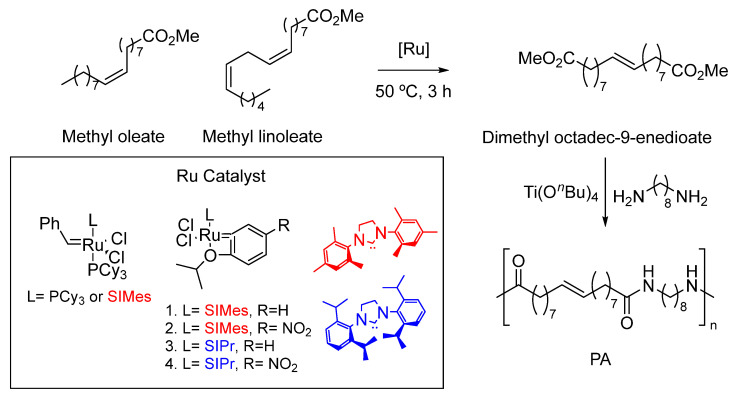
Self-cross-metathesis of technical grade methyl oleate with methyl linoleate using Ru-derived catalyst. Polymerization to obtain semiaromatic PAs.

At this point, it is worth mentioning that obtaining pure methyl oleate is neither easy nor cheap. This makes extrapolation to an industrial scale of procedures developed at the academic level with pure methyl oleate economically unviable. Recently, the use of technical-grade methyl oleate in the synthesis of different polymers has been described by Ortiz and coworkers with the aim of increasing industrial feasibility. In their work, the selective self-cross-metathesis of technical-grade methyl oleate, which contains a significant proportion (approximately 20% by weight) of methyl linoleate, was reported. Using two newly developed Ru-based catalysts, featuring either *N*-heterocyclic carbene ligands or *ortho*-isopropoxybenzylidene moieties, reactions were carried out solvent-free at 50 °C for 3 h. The obtained dimethyl octadec-9-enedioate was polycondensed with diamines or diols using a titanium-based catalyst, without solvent, and under vacuum at elevated temperatures ranging from 150 °C to 200 °C, yielding PEs and PAs. Notably, polycondensation with 1,8-diaminooctane produced a semicrystalline PA with a T_m_ of 152 °C. This example strongly supports the move toward industrial feasibility via accessible feedstocks and solvent-free, energy-moderate processes (Scheme 33) [101].

**Scheme 34:**
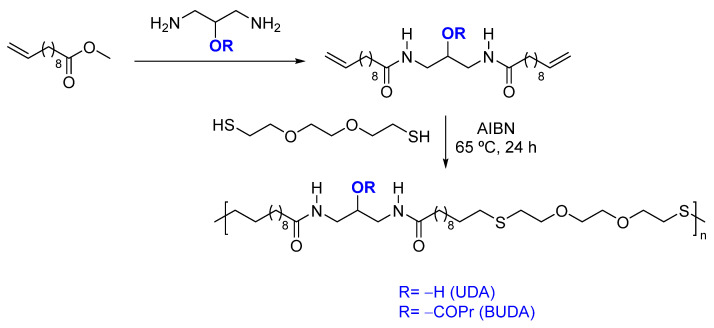
Thiol-ene copolymerization reaction synthesizing PAs with pendant groups, with tunable crystallinity, mechanical performance and photoluminiscence.

Another interesting fatty acid-based PA synthesis starting from methyl undec-10-enoate was reported by C. Tang and coworkers. In this study, the authors reported the synthesis of biomass-derived long-chain PAs bearing pendant polar hydroxyl moieties or, alternatively, non-polar butyrate groups in order to regulate the crystallization behavior of the obtained materials. In this case, the presence of two terminal olefinic double bonds in the co-monomer allowed the homopolymerization and copolymerization reactions to be carried out by a thiol-ene *click* reaction to yield the corresponding functionalized PAs (Scheme 34). The homopolymerized butyrate pendant PA (PBUDA) did not show any melting temperature, but the copolymerized PA displayed melting temperatures ranging from 49.5 °C to 122.3 °C. Indeed, butyrate group restrains the formation of a crystalline structure, enabling an amorphous matrix. However, the hydroxyl group can form a supramolecular hydrogen bonding interaction with side amide/hydroxyl groups, increasing the crystallinity of the PA. Overall, these long-chain PAs exhibited programmable supramolecular architectures with tunable crystallinity and mechanical performance, readily adjustable by varying the co-monomer composition. They studied the mechanical properties of the synthesized non-crystalline homopolymer PBUDA, which exhibited a Young’s modulus of 33.75 MPa. In comparison, the copolymers with pendant hydroxyl groups showed Young’s modulus ranging from 55.6 MPa to 149.6 MPa. The introduction of hydroxyl groups as pendant groups led to an increase in Young’s modulus with increasing content. This suggests that the crystalline structure of the PA component contributed to enhanced material toughness. To further enhance mechanical properties, they decided to introduce metal–ligand coordination into the PAs. Therefore, by coordinating Cu ion with the sulfur atoms, the mechanical strength of these PAs was significantly increased, from 149.5 MPa to 211.2 MPa. Interestingly, the metal-coordinated PAs also provided strong luminescence, particularly the coordinated PAs revealed photoluminescence with emission peaks around 418 nm and UV–visible maximum absorption near 210 nm. While promising, this approach requires precise control of monomer architecture and coordination chemistry, potentially limiting scale-up without further simplification [102].

**Scheme 35:**
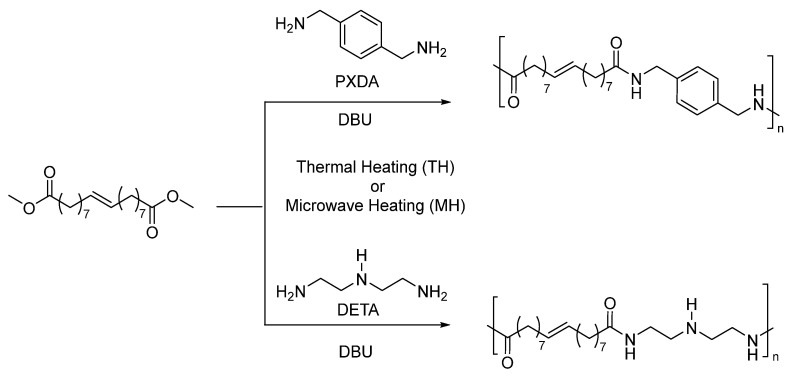
Synthesis of biobased PAs using conventional or microwave heating procedures and starting from dimethyl 9-octadecenedioate.

In the same year, A. Ullah and R. Ahmadi synthesized two bio-based PAs from dimeric FAME precursors using both conventional and microwave-assisted methods. Specifically, dimethyl 9-octadecenedioate was reacted with either *p*-xylylenediamine (PXDA) or diethylenetriamine (DETA), employing DBU as the catalyst (Scheme 35). In all cases, semi-crystalline PAs with high melting temperatures ranging from 45 °C to 201 °C were obtained. The obtained materials revealed degradation temperatures (T_d_^5^) between 124 °C and 373 °C. It has to be highlighted that microwave-assisted synthesis using DETA significantly enhanced crystallinity and melting temperature, reducing reaction time and energy input, compared to its conventionally synthesized counterpart. Further wide-angle X-ray scattering (WAXS) analysis revealed that microwave irradiation facilitated the formation of γ-crystalline phases, while conventional heating predominantly yielded amorphous or α-crystalline structures. Moreover, they studied the mechanical properties of the synthesized PAs, finding that those produced with PXDA exhibited significantly higher tensile strengths, 18.5 MPa and 20.3 MPa using traditional and microwave heating, respectively, compared to the PAs derived from PDETA, which showed much lower values of 1.9 MPa and 0.7 MPa under the same conditions. The increased stiffness of the PXDA-based PAs is likely due to the steric hindrance and rigidity introduced by the aromatic rings in their backbone, in contrast to the more flexible structure of DETA-based PAs. Overall, this study serves as an example of how energy-efficient processing methods can be used to enhance both sustainability and performance in fatty acid-based PAs [103].

**Scheme 36:**
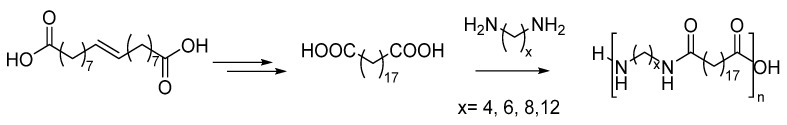
Synthesis of PAs reacting 1,19-nonadecanedioic acid with various aliphatic diamines.

To transition away from the traditional “take, make, dispose” model, the circular economy proposes a regenerative system in which products, materials, and resources are maintained in use for as long as possible, being recovered, recycled, or regenerated at the end of their life, reducing, in consequence, environmental impact and material waste. Addressing this challenge, Greiner and Rist reported the synthesis and chemical recycling of linear aliphatic PAs derived from plant oil-based monomers (Scheme 36). Specifically, 1,19-nonadecanedioic acid, synthesized from oleic acid via a two-step reaction using a Pd catalyst in the initial methoxycarbonylation step, followed by hydrolysis with a KOH/MeOH solution, was subsequently polycondensed with various aliphatic diamines containing 4, 6, 8, or 12 methylene units. The corresponding PA salts were first formed, then melted and subjected to polycondensation under vacuum with gradually increasing temperatures to yield semi-crystalline PAs. GPC revealed high molecular weights ranging from 26 kDa to 51 kDa, with narrow dispersities between 1.6 and 1.9. Thermal characterization via DMA, DSC, and TGA confirmed the formation of semi-crystalline materials with T_g_s between 44 °C and 62 °C, T_m_s from 172 °C to 208 °C, and T_d_s up to 448 °C. Once again, they observed a trend consistent with the previously mentioned examples: increasing the length of the aliphatic chain led to a decrease in the melting temperature. This behavior can be attributed to a reduction in amide group frequency, resulting in less hydrogen bonding interaction. Regarding mechanical properties, the PAs exhibited Young’s moduli ranging from 678 MPa to 959 MPa. The results did not follow a linear trend; the highest modulus was observed for the diamine containing six methylene groups. Both shortening the methylene chain to four or increasing the diamine chain length led to a decrease in Young’s modulus. Remarkably, the authors also demonstrated chemical recycling of the PAs through microwave-assisted hydrolysis in aqueous hydrochloric acid, reducing the reaction time to just 5 min and thereby lowering energy consumption compared to conventional hydrolysis methods. This process enabled the recovery of starting diacid in quantitative yields, which was newly used to synthesize a virgin PA. GPC analysis of the new polymer showed a M_n_ of 38 kDa and enhanced tensile strength and Young’s modulus compared to the original polymer (from 959 MPa to 1370 MPa). Overall, these results underscored the potential for closed-loop recycling, providing a significant advance toward circular bio-based polyamide materials [95].

Overall, the incorporation of fatty acid-based monomers into PA structures not only reduces the reliance on fossil resources but also provides opportunities to tailor polymer performance by varying chain length, degree of unsaturation, and branching. These properties can influence crystallinity, flexibility, and hydrophobicity, making fatty acid-derived PAs suitable for applications ranging from packaging and textiles to automotive and biomedical fields. Additionally, the position of the double bond, the length of the carbon chain, and whether the double bond is internal or terminal can significantly influence the material properties of the resulting polymeric materials [94]. As a summary, Table 5 is presented, compiling all the works described in this section and highlighting the most significant aspects of each.

## 6. Conclusions and Future Trends

Polyamides (PAs) represent a class of polymers with broad applications across diverse sectors, ranging from everyday plastics to high-performance materials used in automotive, textiles, electronics, and biomedical devices. Since their introduction in the 1930s with fossil-derived Nylon, their relevance has continued to grow. In recent decades, the urgent need for sustainable materials has spurred the development of biobased PAs, a new generation of polymers derived from renewable resources, which can reduce carbon emissions and diminish the dependence on fossil stocks. Beyond their environmental benefits, these polymers offer structural diversity and functional versatility due to the rich chemical variety of natural feedstocks. Functional groups, stereochemical configurations, and chain flexibility can be fine-tuned, giving rise to materials with tailored properties for specific applications. There is no doubt, therefore, that much progress has been made in this regard. PAs synthesized from biobased monomers are increasingly versatile and have increasingly improved properties, even surpassing their fossil-based analogues in certain cases.

It is true that the starting substrates are biobased and that the percentages of biogenic carbon in the final polymers are increasing. However, while significant progress has been made, further efforts are required. More sustainable processes with lower energy requirements must be developed, the use of organic solvents must be minimized or even eliminated, and metals must be replaced with more environmentally friendly catalysts or even enzymes. Furthermore, methods for extracting monomers from biosources must also be improved. In this sense, furan derivatives constitute an example. These compounds are usually classified as *sleeping giants*. Despite their enormous synthetic potential, their real applications are relatively limited, mainly due to the difficulty of obtaining them in biorefineries on an industrial scale. While the sustainability potential of these materials is widely acknowledged, a consistent lack of quantitative data, such as life cycle assessments (LCAs), energy consumption, and carbon footprint analyses, remains a limitation in the current literature. Many studies highlight the use of renewable feedstocks and greener synthetic methodologies, but only a few provide robust, quantitative comparisons with fossil-based counterparts. Consequently, environmental benefits are often reported in qualitative terms, which weakens their credibility. To move forward, future research must integrate environmental metrics directly into polymer development.

Finally, the transition toward circular chemistry is essential. Beyond using biobased monomers and environmentally friendly processes, it is imperative that materials are designed for complete recyclability. This includes their ability to degrade into starting materials, which must then be reused to synthesize new, virgin polymers. Thus, closing the loop and ensuring long-term sustainability.

## Figures and Tables

**Table 1:** Furan-based aliphatic PAs and their properties.

Entry	Substrate	Reaction	Properties	Structure	Ref.
**1**	FDCA, DMFDCA, FDCACl	▪ Interfacial solution ▪ Melt polymerization	▪ T_m_: 125–250 °C ▪ T_g_: 70–100 °C ▪ T_d_^max^: 350–450 °C	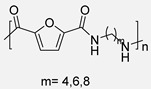	[51]
**2**	DMFDCA	▪ 1,8-octanodiamine ▪ Novozym 435	▪ M_n_: 54 kDa	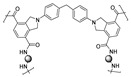	[54]
**3**	FDCA	▪ First prepolymerization step ▪ SSP	▪ T_m_: >280 °C	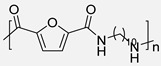	[56]
**4**	FDCACl	▪ Interfacial polymerization	▪ T_g_: 136–280 °C ▪ T_m_: 225–270 °C	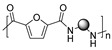	[57]
**5**	DMFDCA	▪ Melt polycondensation ▪ TIPT	▪ M_n_: 8–11 kDa ▪ T_g_: 96–140 °C ▪ T_d_^5^: 395–415 °C ▪ T_d_^max^: 424–446 °C	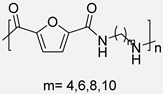	[60]
**6**	DMFDCA	▪ 1,10-decanediamine ▪ TBD	▪ T_g_: 89–142 °C ▪ T_d_^5^: 357–410 °C ▪ T_d_^max^: 435–456 °C ▪ Tensile strength: 52.12 MPa ▪ Tensile Modulus: 1636 MPa ▪ Elongation at break: 69%	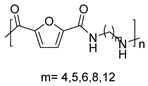	[61,62]
**7**	DMFDCA	▪ Continuous flow technology ▪ TBD	▪ T_g_: 103–131 °C ▪ T_d_^max^:449–465 °C	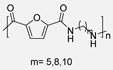	[64]
**8**	DMFDCA	▪ Two-step process ▪ BmimX	▪ M_n_: 27 kDa ▪ T_g_: 150 °C ▪ Elastic modulus: 3.7 GPa	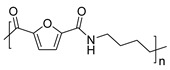	[65]
**0**	DMFDCA	▪ Melt polycondensation	▪ T_g_: 57–104 °C ▪ T_m_: 179 °C ▪ T_d_^5^: 338–412 °C ▪ T_d_^max^:446–473 °C	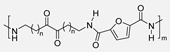	[66]

**Table 2:** Furan-based aromatic PAs and their properties.

Entry	Substrate	Reaction	Properties	Structure	Ref.
**1**	**FDCA**	▪ Direct polycondensation	▪ M_n_: 64–84 kDa ▪ T_g_: 217–302 °C ▪ T_d_^max^: 434–442 °C ▪ Tensile strength: 71.40–94.77 MPa ▪ Tensile Modulus: 998–1635 MPa ▪ Elongation at break: 7–11%	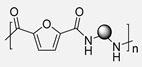	[70]
**2**	**FDCACl**	▪ Melt polycondensation at low temperatures, 70 °C	▪ T_g_: 287–325 °C ▪ T_d_^max^: 424–563 °C	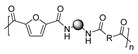	[71]
**3**	**FDCACl**	▪ Low-temperature polycondensation	▪ T_g_: 286 °C ▪ T_d_^max^: 450 °C ▪ Tensile strength: 77.80 MPa ▪ Tensile Modulus: 1134 MPa ▪ Elongation at break: 21%	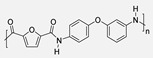	[72]
**4**	**FDCACl**		▪ T_g_: 290 °C ▪ T_d_^max^: 362–640 °C	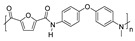	[73]

**Table 3:** Multifuran PAs: synthesis, properties and their structure.

Entry	Substrate	Reaction	Properties	Structure	Ref.
**1**	2,2′-*bis*[2-(5-chloroformyl)furfuryl]propane	▪ Interfacial polymerization	▪ T_m_: 76–117 °C ▪ T_d_^max^: 300–375 °C	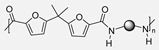	[74]
**2**		▪ Interfacial polymerization	▪ T_m_: 72–120 °C ▪ T_d_^max^: 350–415 °C	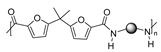	[75]
**3**		▪ Solution polycondensation in NMP	▪ T_g_: 96–125 °C ▪ T_d_^max^: 344–410 °C	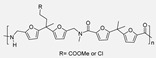	[76]
**4**	Bifuran derived dicarbonylchloride	▪ Solution and interfacial polycondensation	▪ T_d_^5^: 397–408 °C ▪ T_m_: 163–215 °C	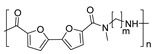	[81]
**5**	Bifurfurylamine	▪ Solution polycondensation	▪ T_d_^5^: 302–325 °C ▪ T_m_: 93–194 °C	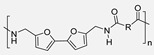	[73]

**Table 5:** Fatty acid-derived PAs.

Entry	Substrate	Reaction	Properties	Structure	Ref.
**1**	Undec-10-enenitrile	Hydroformylation + Oxidation		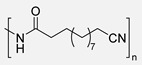	[96]
**2**	Methyl oleate and methyl erucate	▪ Saponification ▪ Catalytic hydrogenation (Ru catalyst) ▪ Polycondensation	▪ T_m_: 152 and 156 °C ▪ M_n_: 10 kDa	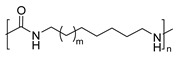	[97]
**3**	Undec-10-enoate, methyl oleate and methyl erucate	1. Thiol-ene *click* Polycondensation with TBD as a catalyst	▪ T_m_: 40–182 °C ▪ M_n_: 3.7–15.9 kDa	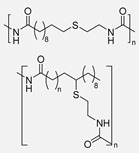	[17]
**4**	Methyl oleate and methyl erucate	Wacker oxidation Reductive amination Polymerization	▪ T_m_: 224–250 °C ▪ M_n_: 11.2–21.4 kDa ▪ T_d_^max^: 350 °C	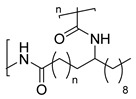	[98]
**5**	Methyl undec-10-enoate, methyl oleate, and methyl erucate	Wacker oxidation Reductive amination Polymerization	▪ T_m_: 70–161 °C ▪ M_n_: 24.4–33.5 kDa	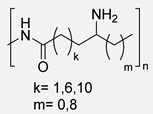	[99]
**6**	Methyl oleate and methyl erucate	Wacker oxidation Reductive amination Lossen rearrangement Cross-metathesis Reductive deprotection Polymerization	▪ T_m_: 169 and 186 °C ▪ M_n_: 14.9–22.6 kDa ▪ Young’s Modulus: 1480–2320 MPa	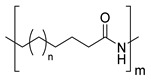	[100]
**7**	Technical-grade methyl oleate	Metathesis 2. Polycondensation	▪ T_m_: 152 °C	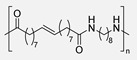	[101]
**8**	Methyl undec-10-enoate	Thiol-ene *click* polymerization	▪ T_m_: 49.5–122.3 °C ▪ T_g_: (−29.4)–(−21.6) °C ▪ Young’s Modulus: 14.8–149.5 MPa	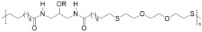	[102]
**9**	Dimethyl 9-octadecenedioate	▪ Conventional method ▪ Microwave-assisted method	▪ T_m_: 45–201 °C ▪ T_g_: 9–78 °C ▪ T_d_^max^: 124–373 °C ▪ Tensile strength: 0.7–20.3 MPa ▪ Slongation at break: 8.0–128.6%	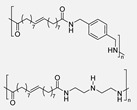	[103]
**10**	1,19-nonadecanedioic acid	▪ Polycondensation	▪ T_m_: 172–208 °C ▪ T_g_: 44–62 °C ▪ M_n_: 26–51 kDa ▪ T_d_^max^: >448 °C ▪ Tensile strength: 36 MPa ▪ Young’s Modulus: 959 MPa ▪ Elongation at break: 169%	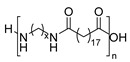	[95]

## Data Availability

The data that support the findings of this study are available from the corresponding author upon reasonable request.

## Abbreviations

The following abbreviations are used in this manuscript:

AROP	Anionic ring-opening polymerization
BHMF	2,5-*bis*(hydroxymethyl)furan
Bmim	1-butyl-3-methylimidazolium
Bz	Benzoylated lactam
CALB	Candida Antartica Lipase B
CC	Cyclocarbonated
CH_2_Cl_2_	Dichloromethane
CROP	Cationic Ring-Opening Polymerization
DFA	Dimer fatty acid methyl esters
DBU	1,8-diazabicyclo[5.4.0]undecen-7-ene
DETA	Diethylenetriamine
DMA	Dynamic Mechanical Analysis
DMF	N,N’-Dimethylformamide
DMFDCA	Dimethoxyferulic dicarboxylate
DMP	Dimethyl pimelimidato
DSC	Differential Scanning Calorimetry
DVA	Divanillinic acid
FAME	Fatty acid methyl ester
FDCA	2,5-Furandicarboxylic acid
FDCACl	2,5-Furandicarboxylic acil chloride
FTIR	Fourier Transform Infrared Spectroscopy
GPC	Gel Permeation Chromatography
GPC/SEC	Gel-permeation chromatography
HMDA	Hexamethylenediamine
HMF	5-hydroxymethylfurfural (HMF)
IR	Infrared Spectroscopy
MALDI	Matrix-Assisted Laser Desorption/Ionization
MDA	1,8-diamino-*p*-menthane
MPC	*m*-phthaloyl chloride
MPD	*m*-phenylenediamine
MULCH	Monounsaturated long-chain
M_n_	Molecular weight
NMR	Nuclear Magnetic Resonance
PA	Polyamides
PA6,6	Poly(hexamethylene adipamide)
PA8F	Polyamide 8 Furan or Poly(octamethylene furandicarboxamide)
PA8T	Poly(octamethylene terephthalamide)
PFA	polyfluoroalkyl
PPD	*p*-phenylenediamine
PXDA	*p*-xylylenediamine
Ref.	Reference
ROP	Ring-opening polymerization
SEM	Scanning Electron Microscopy
SSP	Solid-state polymerization
TBD	1,5,7-Triazabiciclo[4.4.0]dec-5-ene
TCL	Terephthaloylchloride
T_d_	Degradation temperature
T_g_	Glass transition temperature
TGA	Thermogravimetric analysis
THF	Tetrahydrofuran
TIPT	Titanium isopropoxide
T_m_	Melting temperature
TPA	Terephthalic derived polyamides
US	United States
UV	Ultraviolet
WAXS	Wide-Angle X-ray Scattering
